# Targeting SRPK3 Attenuates Cardiac Hypertrophy and Heart Failure by Improving Mitochondrial Bioenergetics through mRNA Splicing and Decay

**DOI:** 10.34133/research.1400

**Published:** 2026-08-24

**Authors:** Yu Zhan, Gaoyuan Liang, Jie Wang, Xianfeng Cen, Nan Zhang, Nan Zhao, Yun Xing, Lanlan Li, Tianhang Yu, Wensheng Dong, Wei Deng, Saiyang Xie

**Affiliations:** ^1^ Department of Cardiology, Renmin Hospital of Wuhan University, Wuhan 430060, China.; ^2^ Hubei Key Laboratory of Metabolic and Chronic Diseases, Wuhan 430060, China.; ^3^ Taikang Center for Life and Medical Sciences, Taikang Medical School, Wuhan University, Wuhan 430072, China.; ^4^ Central Laboratory, Renmin Hospital of Wuhan University, Wuhan 430060, China.; ^5^ Department of Cardiovascular Surgery, Lihuili Hospital Affiliated to Ningbo University, Ningbo, Zhejiang 315041, China.

## Abstract

Pathological cardiac hypertrophy is definitely identified as an adverse deterioration of hemodynamically stressful overload, ultimately heightening risk of sudden death as heart failure (HF) ensues. However, molecular mechanisms underlying cardiac hypertrophy pathogenesis have not been fully described. Herein, we employed targeted genetic approaches and in vivo functional imaging to describe a potent cardiac pro-hypertrophic role of serine arginine protein kinase 3 (SRPK3), one of RNA splicing protein kinases family, which serves as a regulator in pathological hypertrophy and HF. Our study revealed heightened level of SRPK3 in the myocardium of transverse aortic constriction (TAC)-administrated mice and patients who suffer from hypertrophic hearts. Adult mammalian extended expression of *Srpk3* in cardiomyocytes (CMs) led to mitochondrial defects and dysfunction, inducing spontaneous concentric hypertrophy and HF, and ultimately premature mortality. Conversely, conditional deletion of *Srpk3* in CMs attenuated TAC-induced mitochondrial deficits, consequently ameliorating cardiac hypertrophy and HF. Mechanistically, in canonical splicing regulation, we identified SRPK3- serine/arginine splicing-rich factor 2 (SRSF2)-mediated RNA alternative splicing affecting genes related to mitochondrial function. Also, a noncanonical role of SRPK3 involving binding to RPS3 at Ser^149^ to destabilize and degrade PGC1α mRNA was unveiled. Using genetically edited human induced pluripotent stem cell-derived cardiomyocytes (hiPSC-CMs), we also showed that SRPK3-induced dual pathway coordinately resulted in defective mitochondrial biogenesis and bioenergetics, thus triggering pathological cardiac hypertrophy, as evidenced by the SRPK3-induced rewiring of mitochondrial proteome and impaired heart fueling. In preclinical treatment, intravein injection of adeno-associated virus 9–*Srpk3* short hairpin RNA attenuated concentric hypertrophy and HF progression in mice. Together, our findings help to elucidate the mechanism of mitochondrial proteome depletion in triggering hypertrophic hearts, establishing SRPK3 suppression as a long-term and improved strategy for treating pathological cardiac hypertrophy and HF.

## Introduction

The adult mammalian heart often remains quiescent and growth-arrested under homeostatic conditions. Nevertheless, postnatal hearts unexpectedly undergo hypertrophic growth primarily when challenged by excess hemodynamic workload [1]. Rather than benefiting from reversible physiological hypertrophy frequently triggered by exercise and pregnancy [2], due to other factors, most individuals could be at risk of developing pathological cardiac hypertrophy. An irreversible and unequivocal ominous escalation of hemodynamic impediments including hypertension and aortic stenosis can potentially lead to heart failure (HF) [3]. Only a minority of HF patients suffer from genetic causes of hypertrophy cardiac myopathy (HCM) [4], and hypertrophic-phenotype cardiopathy is the predominant consequence of hemodynamic instability [5]. Furthermore, most genetic editing strategies are focused on correcting idiopathic hypertrophy [6], limiting their broader application in secondary cardiomyopathies, thus hindering the optimization of HF treatment.

Various aspects of RNA metabolism, from synthesis to degradation throughout the gene transcription and translation processes [7], serve as a marked complement to the central dogma, structurally related to the crosstalk of spliceosomes and ribosomes, and spatially attached to the karyoplasmic nexus [8]. Therapeutic RNA manipulation has evolved to include dynamic RNA modifications, RNA alternative splicing (AS), nucleoplasmic RNA transport, and RNA stabilization and decay, which cooperate in posttranscriptional and translational regulation in eukaryotes [8,9]. Mitochondria, the principal energy suppliers, contain >1,000 proteins, including the overwhelming majority of proteins derived from the nuclear genome but merely 13 proteins encoded by the mitochondrial genome [10]. Although the emergence of the mitochondrial epi-transcriptome has accelerated the exploration of mtRNA modification [11] and linked mtRNA modification to metabolic plasticity [12,13], the precise molecular mechanisms underlying the involvement of nuclear genome-derived RNA (encoding mitochondrial protein) metabolism in adjusting mitochondrial biosynthetic homeostasis and postnatal cardiac growth are yet to be fully elucidated.

Hypertrophic myocardium is ultra-structurally manifested as a disorganized cardiac fiber layout coupled with deficits in mitochondrial biogenesis and bioenergetics, which precede the overt cardiac structural deterioration [14]. As an insidious indicator of hypertrophic-phenotype cardiomyopathy, mitochondrial abnormalities include extensively disintegrated mitochondria, cristae rupture, loss of mtDNA, and impaired mitochondrial assembly, which spark severe compromise of mitochondrial oxidative phosphorylation (OXPHOS), leading to defective cardiac fuel utilization [15,16]. Compounding these defects, aberrant RNA splicing and posttranscriptional dysregulation in RNA stabilization and decay significantly negatively impact mitochondrial contents and circulatory energy metabolism [17,18]. Ultimately, abnormal nuclear genome-derived RNA (encoding mitochondrial protein) metabolism triggers ineffective mitochondrial biogenesis and bioenergetics in CMs, resulting in cardiac energy shortage and postnatal hypertrophic growth, finally leading to cardiac remodeling and HF with an unfavorable prognosis [19].

Serine/arginine protein-specific kinases (SRPKs), a class of protein kinase families with specific phosphorylation splicing factors rich in serine/arginine (SR) proteins, are mainly classified into 3 subtypes: SRPK1, SRPK2, and SRPK3 [20,21]. According to prior publication, SRPKs regulate the nuclear localization of SR proteins, thereby modulating pre-mRNA AS [22,23]. Specifically, unlike broadly distributed SRPK1 and SRPK2, SRPK3 functions as a muscle-specific kinase in skeletal muscle development [20,24,25]. Indeed, enhanced SRPK3 activity in skeletal muscle has been investigated in transgenic rodents using the muscle creatine kinase (MCK) cis-regulatory elements, including promoters and enhancers [26,27]. However, the involvement and mechanism of SRPK3 in regulating postnatal cardiac growth and HF remain poorly understood. Herein, we examined the role of the SRPK3-RNA metabolism axis in the pathogenesis of pathological cardiac hypertrophy, dissected its molecular mechanism of action in genetically edited mice and human induced pluripotent stem cell (hiPSC)-derived CMs, and assessed its potential utility in HF treatment.

## Results

### Highly expressed SRPK3 inside CMs couples to hypertrophic-phenotype cardiopathy development

We initially performed a bioinformatics sub-transcriptome analysis using a mouse model of transverse aortic constriction (TAC) to probe previously uncharacterized cellular RNA metabolic factors that drive pathological cardiac hypertrophy (Fig. S1A and B). We discovered that SRPK3-SRSF2 (serine/arginine splicing-rich factor 2), a canonical RNA splicing axis, was significantly activated in the hypertrophic myocardium (Fig. S1C). Notably, SRSF2, a putative phosphorylated substrate of SRPKs, is well known to play a role in dilated cardiomyopathy and to promote HF progression [25,28]. On the other hand, the specific function of the kinase SRPK3 in the context of cardiac diseases remains poorly understood.

**Fig. 1.**
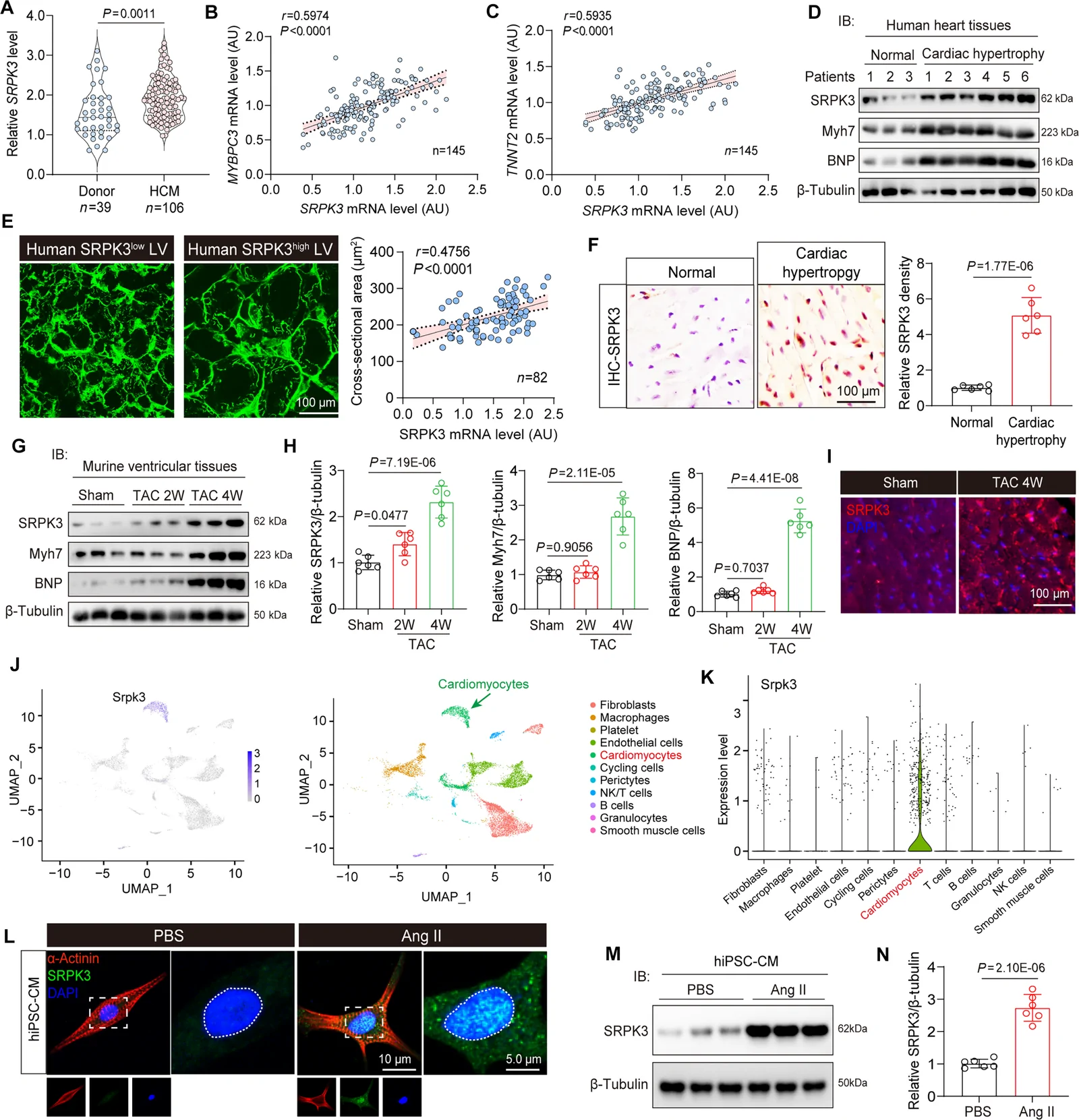
Up-regulation of SRPK3 in hypertrophic hearts. (A) Transcriptional expression of SRPK3 in human RNA-seq data from donor (*n* = 39), HCM (*n* = 106) samples (GSE36961). (B and C) Positive correlation between levels of SRPK3 and different hypertrophic elements (*MYBPC3* and *TNNT2*) among HCM and control tissues, and correlation was analyzed using Spearman correlation analysis. (D) Immunoblot of postmortem LV tissues from 3 control and 6 individuals with definite cardiac hypertrophy. (E) Wheat germ agglutinin (WGA) imaging of postmortem LV frozen sections from SRPK3^high^ or SRPK3^low^ HF individuals, and positive correlation between SRPK3 mRNA and CSA of myocytes in LV, and correlation was analyzed using Spearman correlation analysis. (F) Representative IHC images of SRPK3 in human LV paraffin sections of normal individuals (*n* = 6) and cardiac hypertrophy individuals (*n* = 6). (G and H) Immunoblot of murine LV tissues from sham (*n* = 6), TAC 2 week (*n* = 6)-treated and TAC 4 week (*n* = 6)-treated animals, and relative levels of SRPK3, Myh7, and BNP. (I) Representative IF images of SRPK3 in LV paraffin sections from TAC 4 week-treated mice. (J) Uniform manifold approximation and projection (UMAP) plot showing 11 annotated cell types based on top enriched marker genes in the snRNA-seq data from the C57BL/6 mouse heart (left). Feature plot showing Srpk3 scaled expression across the whole cell clusters. Blue dots labeling cells expressing Srpk3 with up to 95% of the maximum level, and the top 5% high expressing cells were filtered in case of any unknown bias (right). (K) Violine plot showing Srpk3 scaled expression across the whole cell types in the mouse heart snRNA-seq data as shown in (J). (L) Representative IF images of SRPK3 in hiPSC-CMs. (M and N) Immunoblot and relative levels of SRPK3 in hiPSC-CMs treated with PBS (*n* = 6) or Ang II (*n* = 6). *r* and *P* values of the Pearson correlation coefficient are shown. Unpaired Student’s *t* test was used for 2-group comparisons, and one-way ANOVA with an LSD post hoc test was used for multi-group comparisons.

Human multiorgan transcriptomics identified the heart as the main SRPK3 protein-expressing organ (Fig. S1D). Furthermore, among various tissues from adult mice, SRPK3 was predominantly enriched in myocardial tissue and skeletal muscle (Fig. S1E to G), confirming its intramuscular distribution. Although SRPK3 was weakly expressed in the heart under normal conditions, it was highly up-regulated in human hypertrophic hearts (Fig. 1A and Fig. S2A), coinciding with the reduction in SRPK1 and SRPK2 transcription (Fig. S2B and C). Additionally, human cardiac SRPK3 transcript levels and hypertrophic markers such as *MYBPC3*, *TNNT2*, and *TPM1* equivalently showed a certain degree of positive correlation (Fig. 1B and C and Fig. S2D). To establish whether SRPK3 and Myh7 are jointly up-regulated in HF hearts, we compared their protein expression in heart tissues from 47 HF individuals and 35 age- and sex-matched control subjects with no known cardiac disease (Fig. S3A and Table S1). Intriguingly, SRPK3 protein expression was significantly enhanced in tissues from HF patients compared with control tissues, which positively correlated with Myh7 elevation (Fig. S3B to D). Furthermore, immunoblotting confirmed the elevated SRPK3 protein expression in the hypertrophic hearts of HF patients, along with increased Myh7 and brain natriuretic peptide (BNP) expression (Fig. 1D and Fig. S3E). Notably, compared with SRPK3^Low^ left ventricle (LV), SRPK3^High^ LV in HF patients exhibited CMs with an enlarged cross-sectional area (CSA), which was accompanied by an enhanced SRPK3 mRNA expression (Fig. 1E). Furthermore, immunohistochemistry (IHC) analysis confirmed these findings (Fig. 1F). In parallel, SRPK3 was also abundantly expressed in myocardial tissue specimens from rodents with TAC surgery for 4 weeks (Fig. 1G and H), consistent with the results of SRPK3 immunofluorescence (IF) in mouse hearts (Fig. 1I). Single-nucleus RNA sequencing (snRNA-seq) analysis revealed that CMs are the primary Srpk3 mRNA-expressing cell type in adult mouse cardiac tissue (Fig. 1J and K and Fig. S4A and B). Additionally, snRNA analysis uncovered the distinctive cellular distribution of SRPK3 in human hypertrophic hearts [29] (Fig. S4C). Among cellular subpopulations of hearts from adult mice, angiotensin II (Ang II), an impactful hypertrophy-elicited factor, merely sparked Srpk3 up-regulation in CMs (Fig. S4D and E). This outcome prompted us to focus on SRPK3 expression within CMs; hence, hiPSC-induced cardiomyocytes (hiPSC-CMs) were utilized in the follow-up experiment (Fig. S4F). As shown by confocal microscopy, SRPK3 was weakly stained in hiPSC-CMs cultured under normal condition but was substantially enhanced in Ang II-treated hiPSC-CMs (Fig. 1L), in line with immunoblotting outcomes (Fig. 1M and N). Notably, as evidenced by the characteristic SRPK3 protein aggregation in the nucleus rather than in cytoplasmic components (Fig. S4G), SRPK3 enrichment was predominantly enriched in the nuclear range (Fig. 1L).

To identify the disparate consequences of SRPK3 in physiological cardiac hypertrophy, we constructed a mouse model of endurance swimming training to mimic physiological cardiac growth, characterized by enhanced hemodynamic demands (Fig. S5A to F), and adaptive hypertrophy (Fig. S5G and H). Intriguingly, the exercise group of mice showed no difference in SRPK3 protein levels (Fig. S5I and J), which was equivalent to hiPSC-CMs receiving insulin-like growth factor 1 (IGF-1) (Fig. S5K and L), a neurohumoral factor provoking cardiac adaptive growth [30,31]. Coincidentally, previous publicly available dataset (GSE56348 and GSE73896) also supported the comparative SRPK3 expression between physiological stimuli and normal conditions (Fig. S5M and N). These findings substantiate the notion that increased SRPK3 expression in CMs is a hallmark of pathological cardiopathy rather than a feature of reversible cardiac hypertrophy.

### Genetic manipulation of cardiac Srpk3 using CRISPR-Cas9 base editing technologies controls pathological cardiac hypertrophic phenotype

**Fig. 2.**
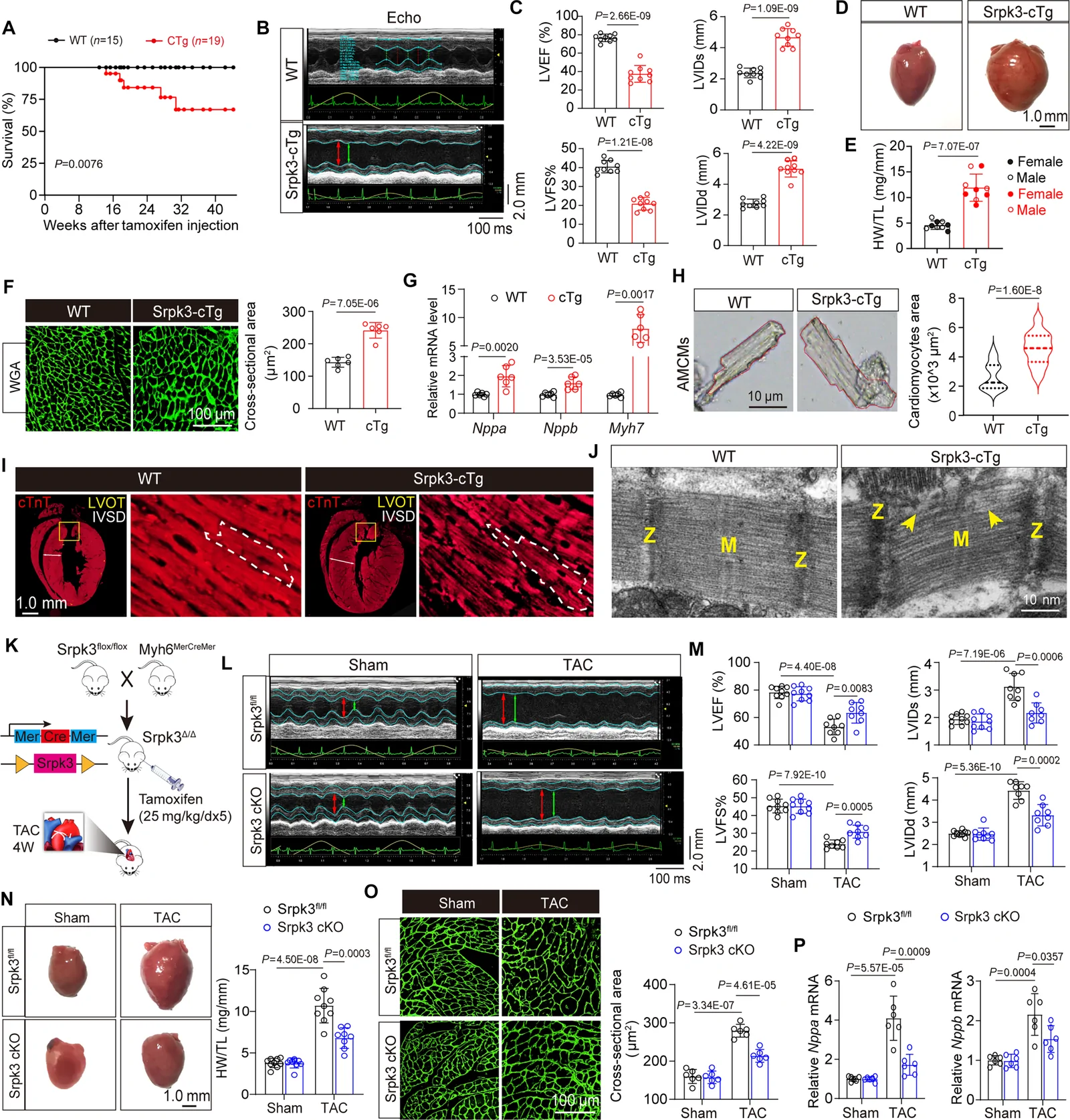
Genetic SRPK3 manipulation regulates concentric hypertrophy. (A) Kaplan–Meier plot showing increased death in adult *Srpk3* cTg mice after tamoxifen induction. (B to J) Assessment of WT and Srpk3 cTg hearts at 6th week after tamoxifen induction. (B) M model echocardiography of mice. (C) Echocardiography parameters of WT group (*n* = 9) and SRPK3 cTg (*n* = 9) including LV ejection fraction, LV fractional shortening, and LV end systolic/diastolic diameter of (B) were calculated. (D) Representative anatomic images of mice. (E) Heart weight/tibia length (HW/TL) ratio of WT group [*n* = 9, 5 males (M) and 4 females (F)] and Srpk3 cTg (*n* = 9, 4 M/5 F) was calculated. (F) Representative WGA images and quantification of CSA of WT (*n* = 6) and Srpk3 cTg (*n* = 6) myocytes. (G) Relative mRNA of hypertrophic factors of myocardial homogenate from WT (*n* = 6) and Srpk3 cTg (*n* = 6) mice. (H) Representative isolated adult mouse CMs (AMCMs) from indicated mice, and CSA was calculated (*n* = 114,WT; *n* = 131,cTg). (I) Representative IF images of cTnT (red) in the myocardium, and outline of individual CMs (white dotted border) and LV outflow tract (yellow frame) with interventricular septal thickness (white arrow) were annotated. (J) Ultrastructure of sarcomere (Z and M line) was presented with TEM, and defective myofilament (yellow arrow) was annotated. (K) Schematical map of Srpk3 cKO mice subjected to TAC surgery. (L to P) Assessment of WT and Srpk3 cKO hearts with sham or TAC for 4 weeks. (L). M model echocardiography of mice. (M) Echocardiography parameters of WT (*n* = 9) and Srpk3 cTg (*n* = 9) including LV ejection fraction, LV fractional shortening, and LV end systolic/diastolic diameter of (L) were calculated. (N) Representative anatomic images of mice, and HW/TL ratio of Srpk3^fl/fl^ (*n* = 9) and Srpk3 cKo (*n* = 9) subjected to sham and Srpk3^fl/fl^ (*n* = 9) and Srpk3 cKo (*n* = 9) subjected to TAC was calculated. (O) Representative WGA images and quantification of CSA of myocytes. (P) Relative mRNA of hypertrophic factors of myocardial homogenate from indicated mice of Srpk3^fl/fl^ (*n* = 6) and Srpk3 cKo (*n* = 6) subjected to sham and Srpk3^fl/fl^ (*n* = 6) and Srpk3 cKo (*n* = 6) subjected to TAC. *P* values were shown. Two-tailed Student’s *t* test was used for 2-group comparisons, and one-way ANOVA with an LSD post hoc test was used for multi-group comparisons.

The mechanistic basis underlying SRPK3 up-regulation in cardiac tissue, and its contribution to pathological remodeling, remains largely elusive. Therefore, we incipiently generated cardiac Srpk3 transgenic mice model to investigate this phenomenon. However, the overwhelming majority of these transgenic mice died prematurely (within a month), exhibiting a severe concentric hypertrophy and growth retardation. Furthermore, only 3 F0 mice survived to sexual maturity but were infertile, precluding us from establishing cardiac Srpk3 stable transgenic lines. Subsequently, we constructed a mouse model with the conditional transgenic expression of Srpk3 through the utilization of a CRISPR-Cas9 base Rosa26 locus-knock-in approach (*Srpk3*^Tg/Tg^ mice; Fig. S6A). We then crossed *Srpk3*^Tg/Tg^ mice with mice expressing Cre recombinase from the CM-specific *Myh6* promoter (*Myh6*-Cre mice) loaded with the gene encoding the tamoxifen-sensitive estrogen receptor variant to generate *Srpk3*^Tg/Tg^*Myh6*^MerCreMer^ (hereafter referred to as Srpk3 cTg) mice (Fig. S6A to C). These processes made the adult cardiac Srpk3 investigation a reality. Freshly isolated CMs extracted from the Srpk3 cTg strain indeed represented highly expressed Srpk3, and expressed a FLAG-tagged protein without sex-dependent difference (Fig. S6D to F). Intriguingly, Srpk3 cTg mice experienced adolescent mortality after tamoxifen injection (Fig. 2A).

Prolonged Cre overexpression could provoke toxicity inside CMs [32], and systemic tamoxifen enrichment is linked with cardiomyopathy and metabolism frailty in mice [33,34]. Herein, we intracorporally explored the potential cardiotoxicity of *Myh6*-Cre and tamoxifen, and found that body weight, cardiac morphology, and contractile function were normal in 5-d consecutive tamoxifen (25 mg/kg/d)-treated adult C57BL/6J or *Myh6*-Cre mice at the age of 6 months (Fig. S6G). These findings were consistent with previous results observed in constitutive *Myh6*-Cre animals [35], implying minimal or no contribution of *Myh6*-Cre and tamoxifen-mediated toxicity to heart dysfunction in SRPK3 transgenic mice.

To determine whether these animals died of substantial cardiac remodeling, as observed in the aforementioned cardiac Srpk3 transgenic mice, we next evaluated cardiac structural and functional parameters in Srpk3 cTg mice 6 weeks after tamoxifen injection. Echocardiographic abnormalities in Srpk3 cTg mice at the sixth week of induction were suggestive of heart failure with reduced ejection fraction (HFrEF), as evidenced by a prominent reduction in systolic function indices and incremental left ventricular residual volume in the LV (Fig. 2B and C and Table S2). Generally, Srpk3 cTg mice exhibited a higher heart weight/tibia length (HW/TL) ratio without sex-dependent differences (Fig. 2D and E), an elevated CM CSA (Fig. 2F), and an enhanced cardiac fetal gene transcription (Fig. 2G) and proteins levels (Fig. S6H and I), suggesting spontaneous cardiac hypertrophy following Srpk3 up-regulation. Notably, freshly isolated primary CMs from Srpk3 cTg mice had a significantly increased CSA (Fig. 2H). Parallel to these findings, histological analysis revealed a reduction in myocardial fiber number, marked thickening of interventricular septum (IVS), and prominent left ventricular outflow obstruction, all of which were collectively indicative of the concept of hypertrophic-phenotype cardiopathy (Fig. 2I). Ultra-structurally, extended SRPK3 expression disturbed the sarcomere arrangement (Fig. 2J), thereby compromising the well-designed structural framework for cardiac contraction. In in vitro assay (Fig. S7A and B), hiPSC-CMs overexpressing SRPK3 exhibited higher cardiac fetal gene transcription (Fig. S7C), while immunoblotting equally showed a pronounced fetal protein abundance in an SRPK3-dependent fashion (Fig. S7D to F). Similar to the spontaneous concentric hypertrophy phenotype CM observed in Srpk3 cTg mice, hiPSC-CMs expressing SRPK3 exhibited an enlarged cellular area (Fig. S7G and H). Indeed, enlarged CMs, along with myofiber disarray (Fig. S7I), exacerbated maladaptive cardiac hypertrophy and poor systolic function in Srpk3 cTg strain.

To further explore whether the loss of Srpk3 function protects against adult hearts with excess hemodynamic workload, we subjected mice with conditional Srpk3 deletion to TAC surgery (Fig. 2K) [36]. Notably, down-regulation of the murine *Srpk3* gene is relevant with significant cardiac hypoplasia and embryonic lethality at approximately embryonic day 9.5 (E9.5) [37]. Therefore, the animal strain with conditional cardiac Srpk3 deletion (*Srpk3^flox/flox^/Myh6-*MerCreMer, hereinafter referred to as Srpk3 cKO) were constructed using CRISPR-Cas9 base genetic editing and the *Myh6*-MerCreMer-inducible system (Fig. S8A). Disruption of Srpk3 was verified through genetic identification and protein profiling (Fig. S8B to D). Impressively, following TAC treatment for 4 weeks, the animals exhibited a significant reduction in LV ejection fraction (LVEF) and LV fractional shortening (LVFS), as well as increased LV internal diameter at the end-systole (LVIDs) and LV internal diameter at the end-diastole (LVIDd). However, these alterations were reversed upon CM-specific deletion of Srpk3 (Fig. 2L and M and Table S3). Cardiac macroscopic morphology revealed that Srpk3 cKO clearly alleviated hypertrophied myocardium in TAC mice (Fig. 2N), as evidenced by the reduced HW/BW ratio with no obvious difference in the LW/TL ratio (Fig. S8E). Additionally, cardiac histological imaging and isolated adult CM area evaluation collectively revealed that loss of Srpk3 function suppressed cardiac enlargement following stress overload (Fig. 2O and Fig. S8F and G) and optimized cardiac fetal gene transcription in Srpk3 cKO animals (Fig. 2P). In response to Ang II stimulation, SRPK3-silencing hiPSC-CMs (Fig. S9A and B) exhibited a lower cardiac fetal gene expression (Fig. S9C). Furthermore, Srpk3 cKO consistently attenuated the Ang II-induced up-regulation of cardiac growth proteins (Fig. S9D and E). Consistent with the ameliorated hypertrophic phenotype in Srpk3 cKO animals, hiPSC-CMs edited with SRPK3 short hairpin RNA (shRNA) exhibited a reduced cellular area (Fig. S9F and G). These findings indicate that blocking SRPK3 exerts a protective effect against the secondary cardiac hypertrophic phenotype.

### Genomic editing of Srpk3 governs mitochondrial biogenesis and bioenergetics

**Fig. 3.**
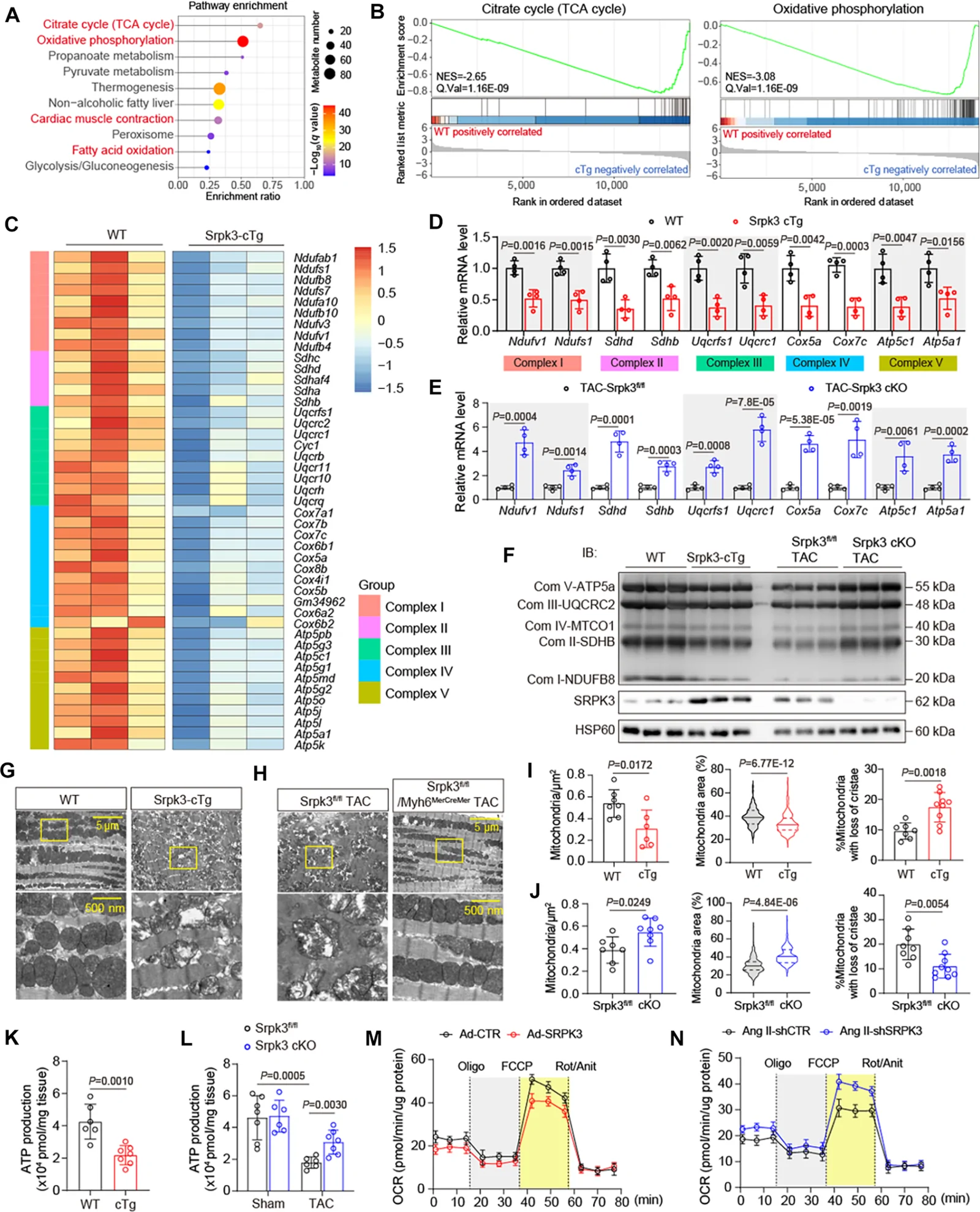
SRPK3 controls overall mitochondrial biogenesis, thereby regulating morphology and function. (A) Gene Ontology terms associated with the 1,181 genes down-regulated in mouse hearts from Srpk3 cTg mice compared to WT mice. (B) GSEA analysis showing the enrichment score of citrate cycle and oxidative phosphorylation gene sets in LV tissues from Srpk3 cTg mice at 6th week of tamoxifen induction or control mice. (C) Heatmap showing differentially expressed mitochondrial respiratory electron transport chain (ETC; complexes I to V) genes in Srpk3 cTg and WT hearts at 6th week of tamoxifen injection. (D) Relative mRNA expression of ETC complex genes in Srpk3 cTg (*n* = 4) and WT (*n* = 4) hearts at 6th week of tamoxifen injection. (E) Relative mRNA expression of ETC genes in WT (*n* = 4) and Srpk3 cKO (*n* = 4) hearts after sham or TAC surgery. (F) Immunoblot of OXPHOS complex in murine LV tissues from Srpk3 cTg and WT mice at 6th week of tamoxifen injection, and WT and Srpk3 cKO mice after TAC treatment for 4 weeks. (G) Representative TEM images of WT and Srpk3 cTg hearts, and (H) WT and Srpk3 cKO mice after TAC operation. (I) Quantification of mitochondria-related parameters including mitochondria/μm^2^ of Srpk3^fl/fl^ (*n* = 7) and Srpk3 cTg (*n* = 6), mitochondria with loss of cristae of WT (*n* = 7) and Srpk3 cTg (*n* = 9), and mitochondrial area from (G). Mitochondria/μm^2^ refers to the average number of mitochondria; mitochondrial area refers to the ratio of mitochondrial area to image area (WT, *n* = 314; Srpk3 cTg, *n* = 279); mitochondria with loss of cristae refers to the percentage of defective mitochondria in individual samples. (J) Quantification of mitochondria-related parameters including mitochondria/μm^2^ of WT (*n* = 7) and Srpk3 cKO (*n* = 8), mitochondria with loss of cristae of Srpk3^fl/fl^ (*n* = 8) and Srpk3 cKO (*n* = 9), and mitochondrial area from (H). Mitochondrial area refers to the ratio of mitochondrial area to image area (WT, *n* = 283; Srpk3 cTg, *n* = 302). (K and L) Measurement of ATP contents in WT (*n* = 6) and Srpk3 cTg (*n* = 7) hearts, and in sham treated Srpk3^fl/fl^ (*n* = 7) and Srpk3 cKO (*n* = 6) hearts or TAC-treated Srpk3^fl/fl^ (*n* = 6) and Srpk3 cKO (*n* = 7) hearts. (M) Oxygen consumption rate (OCR) in hiPSC-CMs with or without Ad-SRPK3 transfection. (N) OCR in hiPSC-CMs with or without SRPK3 silencing after Ang II incubation. *P* values were shown. Two-tailed Student’s *t* test was used for 2-group comparisons, and one-way ANOVA with an LSD post hoc test was used for multi-group comparisons.

To probe the molecular events underlying concentric hypertrophy, we next evaluated the transcriptome of ventricles at the lethal onset (sixth week after tamoxifen induction) in Srpk3 cTg hearts using RNA sequencing (Fig. S10A). We identified 1,809 differentially expressed genes (DEGs), of which 628 (35%) and 1,181 (65%) were up-regulated and down-regulated, respectively (Fig. S10B and C). Furthermore, unsupervised hierarchical clustering of DEGs revealed that they were clustered based on Srpk3 up-regulation. Notably, depressed genes were primarily enriched for Gene Ontology (GO) terms related to the mitochondrial network, including citrate cycle, OXPHOS, and local metabolic processes (Fig. 3A). Concurrently, Gene Set Enrichment Analysis (GSEA) identified the citrate cycle, OXPHOS, fatty acid oxidation, pyruvate metabolism, and cardiac muscle contraction, as the underrepresented gene set in Srpk3 cTg hearts (Fig. 3B and Fig. S10D). Moreover, transcriptomic profiling revealed a substantial reduction in mitochondrial complexes I to V and other mitochondrial components, including the inner membrane, matrix, and outer membrane (Fig. 3C and Fig. S10E). Analysis of mRNA transcription and protein translation confirmed the down-regulation of mitochondrial components in Srpk3-overexpressing ventricles; however, cardiac genetic Srpk3 knockout attenuated the stress overload-induced reduction in overall mitochondrial content (Fig. 3D to F and Fig. S10F to H). Furthermore, the Srpk3 cTg myocardium exhibited a decreased mitochondria DNA content (Fig. S11A). Moreover, transmission electron microscopy (TEM) revealed that compared with control mice, Srpk3 cTg mice had a lower mitochondrial density in LV CMs and a higher proportion of mitochondria with defective cristae (Fig. 3G and I). In the hemodynamic mouse model, mitochondrial morphology was not altered in the sham group (Fig. S11B and C); however, homozygous Srpk3 knockout in CMs improved the TAC-mediated mitochondrial swelling and destruction of cristae (Fig. 3H and J).

In a previous study, an impaired mitochondrial network has long been recognized as a primary cause of insufficient energy generation [38]. Herein, we first evaluated ATP content in the ventricular myocardium to elucidate the role of Srpk3 in energetic response to hypertrophic stimuli. Myocardial adenosine triphosphate (ATP) was down-regulated in Srpk3 cTg mice in the sixth week after tamoxifen induction (Fig. 3K). As expected, ventricular ATP production was also down-regulated in mice subjected to TAC treatment for 4 weeks; however, ATP decline was notably reversed in Srpk3 cKO hearts (Fig. 3L). Furthermore, hiPSC-CMs overexpressing Srpk3 had a lower basal and maximal respiration than those transfected with a control vector (Fig. 3M and Fig. S11D), Additionally, Srpk3 silencing blunted the respiration decline induced by exogenous Ang II in hiPSC-CMs (Fig. 3N and Fig. S11F), despite the normalized respiration in phosphate-buffered saline (PBS)-incubated hiPSC-CMs (Fig. S11E and F), potentially explaining the dramatic alteration in ventricular ATP production in genetically edited mice. In view of the potential activation of mitophagy as implied by the mitochondrial fragment-induced regulation of genes, we examined the regulation of mitophagy-related proteins in Srpk3 cTg and wild-type (WT) CMs. Intriguingly, no change was observed in the abundance of the major mitophagy-related proteins in CMs overexpressing Srpk3. Among the major mitophagy-related proteins, BNIP3L was the only one showing a statistically significant change in CMs overexpressing Srpk3. However, its increase was marginal, as it barely exceeded the significance threshold, and no other mitophagy biomarkers showed discernible alterations, collectively indicating that mitophagy was not prominently activated (Fig. S11G). Subsequently, we packaged the adeno-associated virus serotype 9 (AAV9)–Tnnt2–mtKeima virus and administered it via intramyocardial injection to ensure Keima protein expression specifically in the mitochondria (mtKeima) of CMs [39]. Abundant mtKeima^+^ puncta were comparably distributed in adult mouse CMs (AMCMs) from Srpk3 cTg and WT mice (Fig. S11H and I). The area of dots with a high 560-nm/440-nm ratio was quantitatively indistinguishable between Srpk3 cTg and WT hearts (Fig. S11J and K). These findings validated the hypothesis that Srpk3 elevation triggered the reduction in mitochondrial content primarily due to impaired mitochondrial biosynthesis rather than through implicated intensive mitophagy or clearance.

### SRPK3 physically cooperates with SRSF2 to modulate the RNA AS of mitochondrial function-related genes

**Fig. 4.**
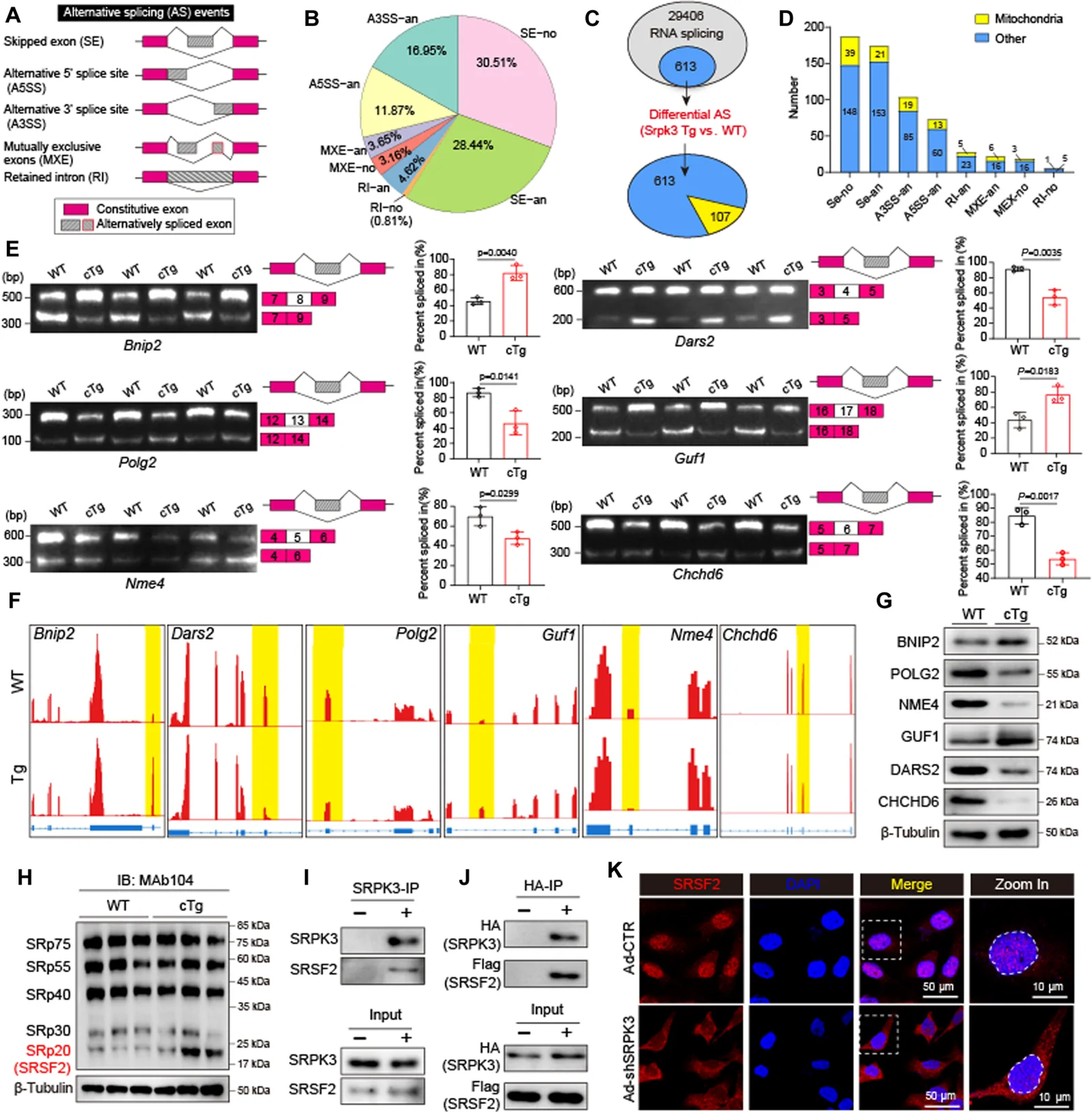
Elevated SRPK3 in CMs causes aberrant AS of mitochondria-related genes by coupling with SRSF2 inside nuclear localization. (A) Summary of differential splicing events using high-throughput RNA-seq analysis in WT and Srpk3 cTg hearts at 6th week of tamoxifen induction. (B) Presentation of all pre-mRNA splicing events identified in the RNA splicing analysis. (C) Pie chart showed the SE events between WT and Srpk3 cTg mice and percentage of mitochondrial related events. Totally, 29,406 SE events were identified, in which 613 of them were differentially changed. One hundred seven of the 613 changed RNA splicing events occurred in genes annotated in mitochondria. (D) Qualitative presentation of each AS event types and the percentage of genes involved in mitochondria between WT and Srpk3 cTg hearts. (E) RT-PCR analysis for mitochondrial genes between WT (*n* = 3) and Srpk3 cTg (*n* = 3) CMs. The middle panels represent the schematic diagram of indicated AS exons. Right panels show the quantification of percent spliced in (PSI). Three independent biological replicates are presented. (F) RNA-seq results of alternative sites in mitochondrial genes using IGV software analysis. (G) Immunoblot analyses of the expression of mitochondrial proteins in WT and Srpk3 cTg CMs. (H) Immunoblot analysis of SR protein phosphorylation by a pan-phospho-SR antibody (MAb104). (I to J) IP and immunoblot analysis of the indicated proteins in hiPSC-CMs (left) and HEK293T cells transfected with Flag-SRSF2 or HA-SRPK3 (right). (K) Representative IF images of SRSF2 (red) in hiPSC-CMs transfected with Ad-shSRPK3 or control. *P* values were shown. Unpaired Student’s *t* test was performed.

Herein, SRPK3, which has been linked with neurodegeneration and skeletal muscle degeneration [30,40], putatively served as a novel interacting partner of RNA AS machinery. This prompted us to establish whether gene-splicing events were affected in CMs overexpressing Srpk3. As expected, RNA-seq profiling revealed numerous mRNA splicing changes in both Srpk3 cTg and WT hearts (Fig. S12A and B), including skipped exons (SEs), alternative 5′ splice sites (A5SSs), alternative 3′ splice sites (A3SSs), mutually exclusive exons (MXEs), and retained introns (RIs) (Fig. 4A). The rMATS software was then applied to detect exon-centric differential splicing events with the default parameters [41]. We analyzed qualitative and quantitative results for both annotated and novel splice junctions, including a summation of 49,883 splicing events for all 5 primary types of AS patterns, with SE as the predominant splicing type, accounting for approximately 58.95% of the AS events affected by Srpk3 up-regulation (Fig. 4B). Furthermore, 613 distinct AS outcomes were identified between Srpk3 cTg and WT hearts. Strikingly, ~17% of the splicing alterations mapped to genes involved in mitochondrial subcellular targeting based on the COMPARTMENTS database (Fig. 4C and D) [42], implying the engagement of SRPK3 in the AS of mitochondrial function-associated genes.

Given that a substantial portion of AS genes annotated in mitochondrial localization varied in hearts overexpressing Srpk3, we sought to establish whether the splicing levels of the corresponding exons of these mitochondrial function-related genes were altered in CMs isolated from WT or Srpk3 cTg mice. AS genes closely associated with mitochondria were selected from DEGs and evaluated through reverse transcription polymerase chain reaction (RT-PCR) using exon-specific primers. According to the results, cells overexpressing Srpk3 showed substantial proportion of SE splicing variants (Fig. 4E), confirming the relative reliability of the RNA-seq data (Fig. 4F). Moreover, immunoblot analysis revealed a congruent alteration in corresponding protein levels of those mitochondrial genes in cells overexpressing Srpk3 compared with their controls (Fig. 4G). SRPK3 overexpression drove AS changes in the majority of mitochondrial genes, together with concomitant decline in mitochondrial proteins levels, largely explaining the reduction of overall mitochondrial content. Notably, the SE splicing variants of Bnip4 and Guf1 are probed to be abnormal, warranting comprehensive follow-up studies.

According to prior work, SRSF splicing factors are putatively involved in AS events [43], and canonical SRPK-SRSF coupling has been verified in RNA metabolism [44,45]. As a serine/arginine kinase, SRPK3 may regulate the RNA AS mediated by SRSF splicing factors through phosphorylation. In this regard, we examined whether SRPK3 could regulate SRSF phosphorylation. Immunoblotting with the MAb104 antibody revealed that compared with those of WT hearts, Srpk3 up-regulation in CMs selectively led to SRSF2 hyperphosphorylation, as evidenced by a pan-phospho-SR antibody immunoblots (Fig. 4H). Furthermore, we validated the physical SRPK3-SRSF2 interaction in hiPSC-CMs and Flag-SRSF2/HA-SRPK3-transfected HEK293T cells (Fig. 4I and J). Meanwhile, SRPK3-SRSF2 binding correlated with increased transcriptional filing in hypertrophic hearts (Fig. S1C). Furthermore, the splicing factors, SRSFs, were translocated to the nucleus and phosphorylated by the SRPK family to exert the pre-mRNA AS effect [44]. Based on the Ang II-induced nuclear enrichment of SRPK3 in CMs, we explored the mechanism of action of local SRPK3 in triggering nuclear SRSF2 assembly in hiPSC-CMs and discovered that CM silencing SRPK3 exhibited a significant decline of nuclear SRSF2 presence, as evidenced by the intensive SRSF2 fluorescence in 4′,6-diamidino-2-phenylindole (DAPI) background (Fig. 4K). These findings support the hypothesis that canonical SRPK3-SRSF2 coupling may promote the AS of mitochondrial genes, thereby partly accounting for myocardial mitochondrial impairment, energy deficiency, and cardiac hypertrophy.

### SRPK3 binds to and phosphorylates RPS3^Ser149^

**Fig. 5.**
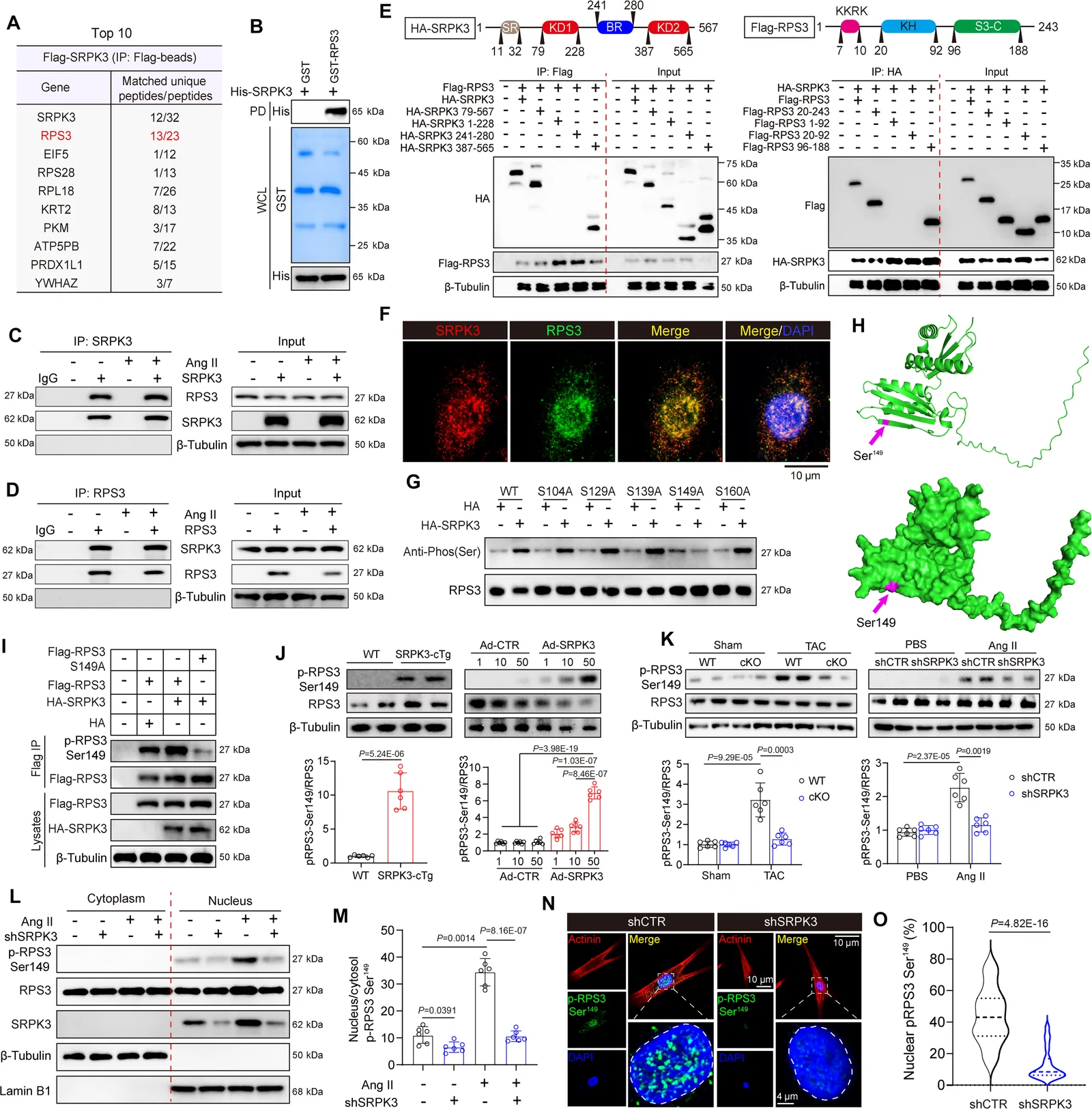
SRPK3 binds to and phosphorylates RPS3^Ser149^, thus promoting its nuclear positioning. (A) Tandem affinity purification–mass spectrometry detection of SRPK3-interacting proteins (obtained from Flag-beads pulldown) after HEK293T cells were transfected with Flag-SRPK3. (B) Glutathione S-transferase (GST) pulldown assay with purified His-SRPK3 and GST-RPS3. (C) hiPSC-CMs with or without SRPK3 overexpression were treated with Ang II and subjected to co-IP with anti-SRPK3 antibodies. (D) CMs with or without RPS3 transfection were treated with Ang II and subjected to co-IP with anti-RPS3 antibodies (down). (E) Flag-RPS3, full-length HA-SRPK3, or truncated mutants of SRPK3 were coexpressed in HEK293T cells for co-IP assay with anti-Flag or anti-HA antibody, respectively. (F) Representative IF images of SRPK3 (red) and RPS3 (green) in hiPSC-CMs. (G) Flag-RPS3 WT and mutant proteins were coexpressed with HA-SRPK3 or free HA in HEK293T cells. Phosphorylation of Flag-RPS3 was determined by immunoblot. (H) The Ser^149^ labeled in pink is present on the surface of RPS3-simulated structure. (I) Flag-RPS3 WT and Ser^149^ mutant proteins were expressed in HEK293T cells along with HA-SRPK3 or free HA. Flag-RPS3 WT and mutant proteins were immunoprecipitated from cell lysates, and their phosphorylation was detected using the pSer^149^-RPS3 antibody. (J) Immunoblot and quantification of phosphorylated RPS3 Ser^149^ in Srpk3 cTg (*n* = 6) mice and WT (*n* = 6) littermates, or in hiPSC-CMs with (*n* = 6) or without (*n* = 6) SRPK3 overexpression. (K) WT (*n* = 6) and Srpk3 cKO (*n* = 6) subjected to sham and WT (*n* = 6) and Srpk3 cKO (*n* = 6) subjected to TAC for 4 weeks. hiPSC-CMs with shRNA-targeting SRPK3 (*n* = 6) or scramble negative control (*n* = 6) were treated with Ang II and hiPSC-CMs with shRNA-targeting SRPK3 (*n* = 6) or scramble negative control (*n* = 6) were treated with PBS. Protein lysates were prepared for immunoblot analysis. (L) hiPSC-CMs transfected with shRNA-targeting SRPK3 or a negative control were treated with Ang II. The cellular fraction was harvested for immunoblot analysis. (M) Quantification of (L) referred to the ratio of nuclear p-RPS3/RPS3 to cytoplasmic p-RPS3/RPS3 (*n* = 6 for each groups). (N) hiPSC-CMs with or without SRPK3 knockdown were treated with Ang II and prepared for IF staining of p-RPS3 and cytoskeleton. (O) Quantification of (N) by calculating the ratio of nuclear p-RPS3 fluorescence units to DAPI fluorescence units (*n* = 237,shCTR; *n* = 158, shSRPK3). *P* values were shown. Two-tailed Student’s *t* test was used for 2-group comparisons, and one-way ANOVA with an LSD post hoc test was used for multi-group comparisons.

To decipher the SRPK3 interaction network, we instantaneously transfected HEK293T cells with exogenous Flag-SRPK3/Vector and performed immunoprecipitation–mass spectrometry (IP-MS) (Fig. S13A). A total of 442 proteins were identified to potentially interact with SRPK3, of which 86 candidates (19.46%) that localized to the nucleus were associated with cardiomyopathy, cardiac contraction, and OXPHOS (Fig. S13B and C), which is consistent with the property of nuclear SRPK3 in CMs. Notably, RNA metabolic processes involved in clusters of orthologous groups (COGs) including gene expression, nucleic acid transport, signal transduction, posttranscriptional modification, and ribosome-mediated protein translation probably implied the noncanonical role of SRPK3 (Fig. S13D). Consistent with previous findings (Fig. S13E and Fig. 4I), SRPK3-SRSF2 coupling emerged as a critical node within the overall SRPK3 interaction network. Furthermore, RPS3, a nucleocytoplasmic RNA-binding protein, was identified as the top prey (Fig. 5A and Fig. S13E). Additionally, in vitro pulldown assays performed with purified recombinant proteins demonstrated that SRPK3 directly bound to RPS3 (Fig. 5B). Moreover, an endogenous IP assays on hiPSC-CMs revealed that SRPK3 directly interacted with RPS3 in CMs (Fig. 5C and D), consistent with the results of spatial molecular docking analysis and exogenous co-IP assay in Flag-RPS3/HA-SRPK3-transfected HEK293T cells (Fig. S13F and G). Next, truncation mutants of SRPK3 and RPS3 were constructed for co-IP assays to explore the molecular domains responsible for the physical SRPK3-RPS3 coupling. Indeed, the kinase domain 2 (KD2) of SRPK3 directly bound to the S3-C domain of RPS3 (Fig. 5E). Furthermore, endogenous SRPK3 and RPS3 colocalized in the nucleus of hiPSC-CMs, as visualized by confocal microscopy (Fig. 5F). For the cytoplasm-localized SRPK2, this activity contributes to delta-secretase conjugation, which is highly phosphorylated (Ser^226^) in human Alzheimer’s disease (AD) brains and is tightly correlated with SRPK2 activity [46]. Accordingly, RPS3 bound to the KD2 domain of SRPK3 at the C terminus, and multiple phosphorylated serine residues were identified throughout the RPS3’s S3-C sequence (amino acids 96 to 188) using MS, with Ser^149^ being the most significantly modified residue (Fig. S13H). Separated point mutations that replace serine with alanine (S104A, S129A, S139A, S149A, and S160A) confirmed the modification of the Ser^149^ residue (Fig. 5G). Ser^149^, a highly conserved site across species (Fig. S13I), resides in the linker of RPS3 and the SRPK3 kinase domain, as well as the interface between a β-plated sheet and a reverse turn structure (Fig. 5H). These data suggest that direct phosphorylation of RPS3 by SRPK3 serves as a key posttranslational modification regulating RPS3 function.

We also sought to determine whether SRPK3 could indeed phosphorylate the Ser^149^ of RPS3 and regulate the nuclear positioning of this alteration. By using a phosphorylation site-specific antibody that recognizes phosphorylated Ser^149^ on RPS3, we discovered that Ser^149^ Ala mutation markedly reduced the Ser^149^ phosphorylation of RPS3 in HEK293T cells (Fig. 5I). Moreover, consistent with p-RPS3^Ser149^ up-regulation in hiPSC-CMs transfected with Ad-SRPK3 in a dose-dependent manner, phosphorylated RPS3 (p-RPS3^Ser149^) was up-regulated in Srpk3 cTg hearts (Fig. 5J). On the other hand, Srpk3 suppression significantly repressed RPS3 phosphorylation attributed by the hypertrophic stimuli (Fig. 5K). Although canonical RPS3 coexists in both the ribosome and nucleus, nuclear RPS3 phosphorylation (non-ribosome-free RPS3) serves as the predominant active form [47]. We also assessed the subcellular p-RPS3^Ser149^ distribution and found that targeted SRPK3 silencing mildly suppressed nuclear p-RPS3^Ser149^ levels under non-hypertrophic conditions (Fig. 5L and M). Notably, Ang II substantially triggered p-RPS3^Ser149^ aggregation within the nucleus, although these effects were not observed in hiPSC-CMs with SRPK3 knockdown (Fig. 5L and M). Similarly, SRPK3 suppression down-regulated nuclear p-RPS3^Ser149^ enrichment, as evidenced by cellular fluorescence visualization in CMs (Fig. 5N and O). These findings indicate that SRPK3 binds to and phosphorylates RPS3 at Ser^149^, promoting its nuclear localization in hypertrophic conditions.

### Ser^149^ phosphorylation of RPS3 by SRPK3 regulates PGC1αmRNA decay and stabilization

**Fig. 6.**
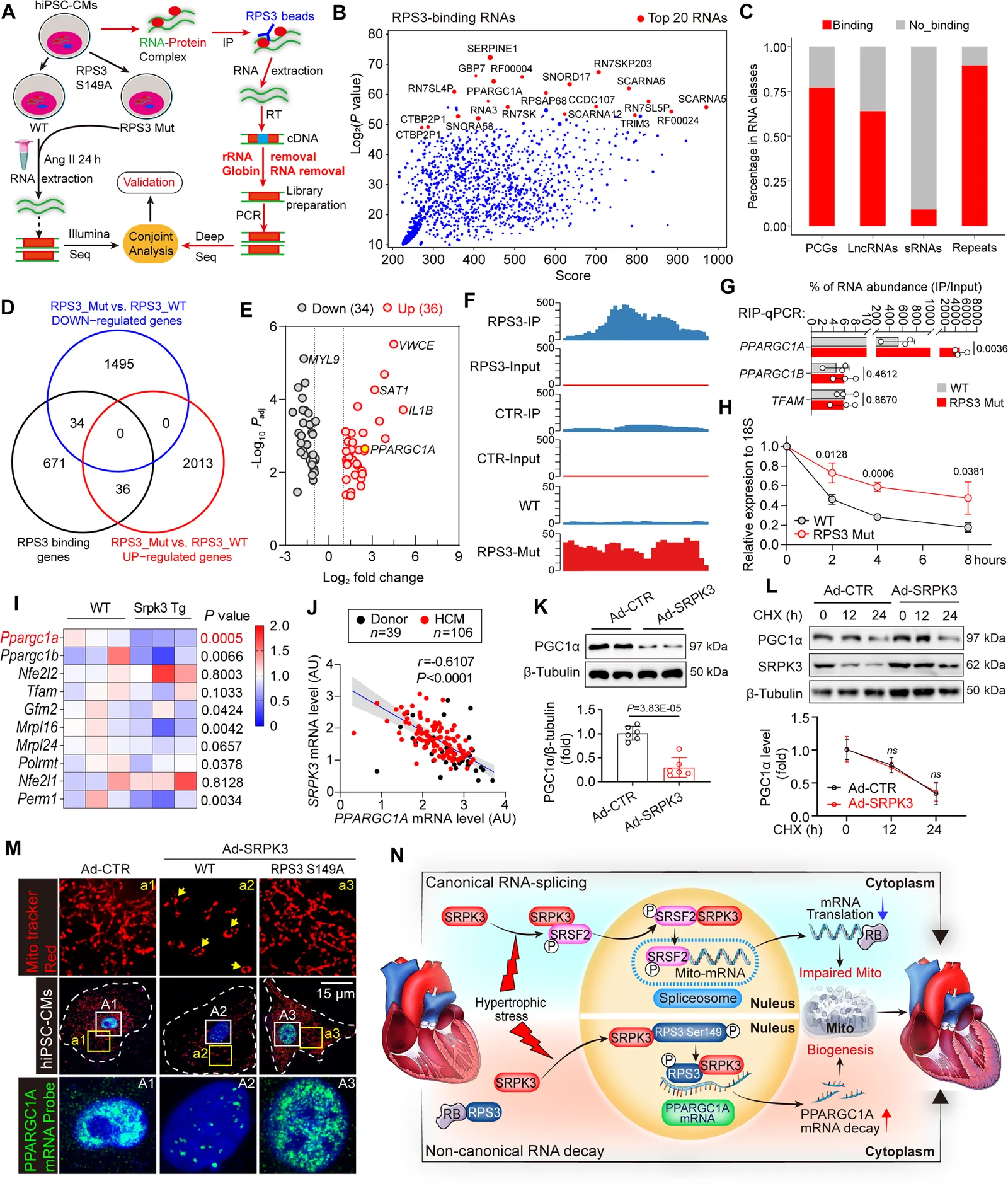
RPS3 physically interacts with PGC1α mRNA and regulates its decay. (A) Scheme for the RPS3 RIP-seq and RNA-seq of Ang II-treated RPS3 WT or Ser^149^ mutant hiPSC-CM experiments. (B) Volcano plot showing RPS3-binding RNAs in hiPSC-CMs. The red dots represent the top 20 targets of RPS3 in CMs. (C) Bar graph showing the RPS3-binding ratios in different RNA classes. (D) Venn diagrams showing the overlap of RPS3-binding protein-coding genes (PCGs) and up/down-regulated genes in RPS3 Ser^149^ mutant CMs. (E) Volcano plots of differential binding proteins in Ang II-treated RPS3 WT or Ser^149^ mutant hiPSC-CMs. (F) Genome browser tracks showing RPS3 RIP-seq peaks and RPS3 Ser^149^ mutant CM RNA-seq peaks at the *PPARGAC1A* locus. (G) RIP-qPCR validation of target binding by RPS3. (H) Measurement of PGC1α mRNA decay in WT and RPS3 Ser^149^ mutant CMs. Samples were collected following transcriptional inhibition using actinomycin D (ActD) for the indicated time. PGC1α mRNA amounts were measured by qPCR according to 18S RNA. (I) Heat map of genes related to mitochondrial biogenesis in prior transcriptome of Srpk3 cTg and WT hearts. (J) Inverse correlation between levels of *SRPK3* and *PPARGAC1A* mRNA among HCM and control tissues (GSE36961), and correlation was analyzed using Spearman correlation analysis. (K) Immunoblot and quantification of PGC1α in CMs subjected to Ad-SRPK3 (*n* = 6) or control (*n* = 6). (L) hiPSC-CMs were infected with Ad-SRPK3 or negative control, and then treated with cycloheximide (CHX; 10 μM) for the indicated time periods. Immunoblot analysis of PGC1α protein levels for each group (Up), and quantification of PGC1α level (Down). (M) Representative images of MitoTracker Red and PGC1α RNA probes inside WT or RPS3 Ser^149^ mutant hiPSC-CMs transfected with Ad-SRPK3 or negative control. (N) Proposed model for the regulation of concentric hypertrophy by canonical SRPK3-SRSF2-mediated RNA AS of mitochondrial function-related genes, as well as RPS3 Ser^149^ binding PGC1α mRNA destabilization and decay, a noncanonical function of SRPK3. RB, ribosome; P, phosphorylation; Mito-mRNA, nuclear genome-derived RNA that encodes mitochondrial protein. *P* values were shown. Two-tailed Student’s *t* test was used for 2-group comparisons, and one-way ANOVA with an LSD post hoc test was used for multi-group comparisons.

We followed these lines of evidence regarding nuclear RPS3’s action to elucidate the mechanism of the SRPK3-RPS3-mediated adverse phenotype. According to prior research, hyperphosphorylated RPS3, which is heavily accumulated in the nucleus (termed as nonribosomal free RPS3), functions as an RNA-binding protein involved in various RNA metabolic processes, similar to an RNA molecular chaperone [48]. This phenomenon could potentially explain the nonsplicing role of SRPK3 in previous protein networks. To further explore the mechanism of SRPK3-mediated RPS3 Ser^149^ phosphorylation in regulating RNA processes, we examined the RNA-RPS3 interaction through RNA immunoprecipitation sequencing (RIP-seq) together with bulk RNA-seq analysis. Specifically, we performed RNA-seq analysis on Ang II-treated RPS3 Ser^149^ Ala mutant or WT hiPSC-CMs and removed ribosomal RNA (rRNA) and globin RNA during DNA library preparation in RIP-seq analysis (Fig. 6A). Consequently, we identified 1,150 transcripts that interacted directly with RPS3 (Fig. 6B). Among them, RPS3-binding elements had the highest mRNA proportion (70.55%), followed by repeat elements (14.77%), long noncoding RNAs (LncRNA) (13.63%), and small nucleolar RNA (snoRNA) (1.05%) in the nucleus (Fig. S14A). We then analyzed the ratio of RPS3-binding RNAs in hiPSC-CMs for their corresponding families and found that both repeat elements and protein-coding genes (PCGs) had high proportion (>75%), indicating that repeat elements and PCGs served as the dominant classes regarding the number of transcripts and RPS3-binding intensity in CMs (Fig. 6C).

To further define the role of RPS3 Ser^149^ phosphorylation in hypertrophic CMs, we analyzed the total RNA-seq data of Ang II-treated RPS3 Ser^149^ Ala mutant (Mut) and WT hiPSC-CMs (Fig. S14B). Among them, 2,234 (39%) and 3,488 (61%) were down-regulated and up-regulated, respectively (Fig. S14C). Interestingly, up-regulated genes were enriched for GO terms related to RNA processes, while down-regulated genes encoded proteins that were mainly associated with GO terms related to routine biological processes (Fig. S14D and E). Moreover, we noticed that RPS3 Ser^149^ Ala mutation markedly enhanced RNA stability in Ang II-treated CMs (Fig. S14F and G). These results were subjected to combined RIP-seq data for repeat elements or gene class analysis by correlating it with high-throughput data, which revealed that one down-regulated repeat element was directly bound by RPS3 (Fig. S14H), whereas 34 of the down-regulated PCGs were bound by RPS3 and 36 of the up-regulated genes showed RPS3-binding properties (Fig. 6D), implying that RPS3 directly bound to and regulated PCGs. Furthermore, up-regulated genes inside RPS3 Ser^149^ Ala mutant hiPSC-CMs were specifically relevant to mRNA stabilization (Fig. S14D). These findings prompted us to focus on the 36 up-regulated PCGs that directly bound to RPS3, of which PGC1α mRNA was the most noticeable in the volcano plot (Fig. 6E) due to its critical roles in mitochondrial biogenesis and bioenergetics [49]. The peaks in the RIP-seq results and fluorescent imaging combined with the RNA probe–fluorescence in situ hybridization (FISH) verified the strong RPS3-PGC1α mRNA interaction (Fig. 6F and Fig. S14I). RIP-qPCR (quantitative polymerase chain reaction) further confirmed the direct binding of RPS3 with PGC1α mRNA (Fig. 6G). Moreover, compared with the WT control, RPS3 showed a markedly higher binding affinity for PGC1α mRNA in Ser^149^Ala mutation, whereas the levels of other mitochondrial genes including *PPARGC1B* and *TFAM*, both of which maintained the same expression in RPS3 mutant CMs, were comparable between groups (Fig. 6G).

The involvement of RPS3 in RNA degradation [50] through nonsense-mediated decay (NMD; a eukaryotic mRNA surveillance system that selectively degrades transcripts) and the association of RPS3 Ser^149^ mutation with RNA stabilization are well-recognized. In this regard, we performed an RNA decay assay to investigate whether RPS3 phosphorylation regulates PGC1α mRNA decay. As expected, PGC1α mRNA decay was mitigated in RPS3 Ser^149^ mutant CMs following Ang II treatment (Fig. 6H). We also observed an enhanced PGC1α mRNA expression in RPS3 Ser^149^ mutant CMs (Fig. S14J), implying a positive correlation between RPS3 phosphorylation and PGC1α mRNA decay. Our previous bulk RNA-seq results on Srpk3 cTg hearts also revealed an essentially reduced *Ppargac1a* transcription (Fig. 6I), and SRPK3 expression was negatively correlated with PGC1α mRNA expression in pathophysiological human hearts (Fig. 6J). Consistent with the transcriptional down-regulation, PGC1α proteins were significantly reduced (Fig. 6K), but this phenomenon was rarely associated with PGC1α degradation (Fig. 6L). Thus, SRPK3 up-regulation-induced RPS3 phosphorylation primarily accelerates PGC1α mRNA decay, a noncanonical RNA destabilization pathway that inhibits mitochondrial biogenesis.

### CRISPR-mediated conversion of nucleus-derived RNA (encoding mitochondrial protein) metabolism impairs hypertrophic growth in SRPK3-overexpressing CMs

To confirm the SRPK3-mediated RNA metabolism (RNA AS, destabilization, and catabolic process) in hypertrophic CMs, we generated hiPSC cells carrying RPS3 Ser^149^ mutation or SRSF2 RS domain (SR proteins are phosphorylated predominantly on serine residues in this domain [51,52] deletion, using the CRISPR-Cas9 system technology based on single-guide RNA approach (Fig. S15A and B and Tables S4 and S5). Using this approach, hiPSC cells were induced to differentiate into CMs [53], properly described as hiPSC-CMs RPS3 S149A and hiPSC-CMs SRSF2ΔRS. The cells were subsequently transfected with Ad-SRPK3 (Fig. S15C). We observed a 50% reduction in transcription of fetal genes and a moderate increase in the expression of PGC1α mRNA after RPS3 mutation and SRSF2ΔRS in SRPK3-overexpressing CMs (Fig. S15D and E). We operated PCR electrophoresis to detect whether the SRSF2ΔRS mutant can reverse the adverse results brought by SRPK3 overexpression, and the mutation at SRSF2ΔRS significantly ameliorated the SE events of mitochondria proteins (Fig. S15F). Analysis of the overall immunoblot demonstrated that exogenous SRPK3 significantly amplified the expression of hypertrophic proteins and reduced PGC1α intensity, as well as compromised mitochondrial complex content in hiPSC-CMs. This effect was partly abrogated by RPS3 S149A mutation and RS domain deficiency in SRSF2 sequence (Fig. S15G and H). Despite that, to identify if RPS3 S149A mutation and SRSFΔRS can restore the mitochondrial function, they were subjected to Ad-SRPK3, together with blank control group, and analyzed with Seahorse, the results revealing their protective effect on mitochondria function (Fig. S15I and J). Moreover, elevated SRPK3 significantly increased the cellular area, which was attenuated in RPS3 S149A and SRSF2ΔRS CMs, based on the findings from the cytoskeleton immunofluorescent imaging (Fig. S15K and L). These data indicate that genetic disruption of RNA decay and AS process effectively attenuates SRPK3-mediated hypertrophic phenotype in CMs.

PGC1 nuclear localization signal has been reported to be a major driver of mitochondrial biogenesis and functions [54]. Surprisingly, cellular SRPK3 significantly decreased nuclear PGC1α mRNA accumulation and increased mitochondrial fragmentation and “donut” formation (Fig. 6M), a feature of abnormal fission and defective mitochondria [55,56]. However, disruption of the RNA degradation by RPS3 S149A variant restored nuclear PGC1α mRNA aggregation and attenuated formation of donut-shaped impaired mitochondria in CMs (Fig. 6M). Collectively, these results demonstrate that SRPK3 drove nuclear genome-derived RNA (encoding mitochondrial protein) metabolism in cardiac hypertrophic growth, by orchestrating AS of mitochondrial genes and RPS3 Ser^149^ phosphorylation-mediated PGC1α mRNA decay (Fig. 6N).

### Prophylactic benefits of SRPK3 inhibition by AAV9-mediated shSRPK3 delivery in established concentric myocardial hypertrophy

**Fig. 7.**
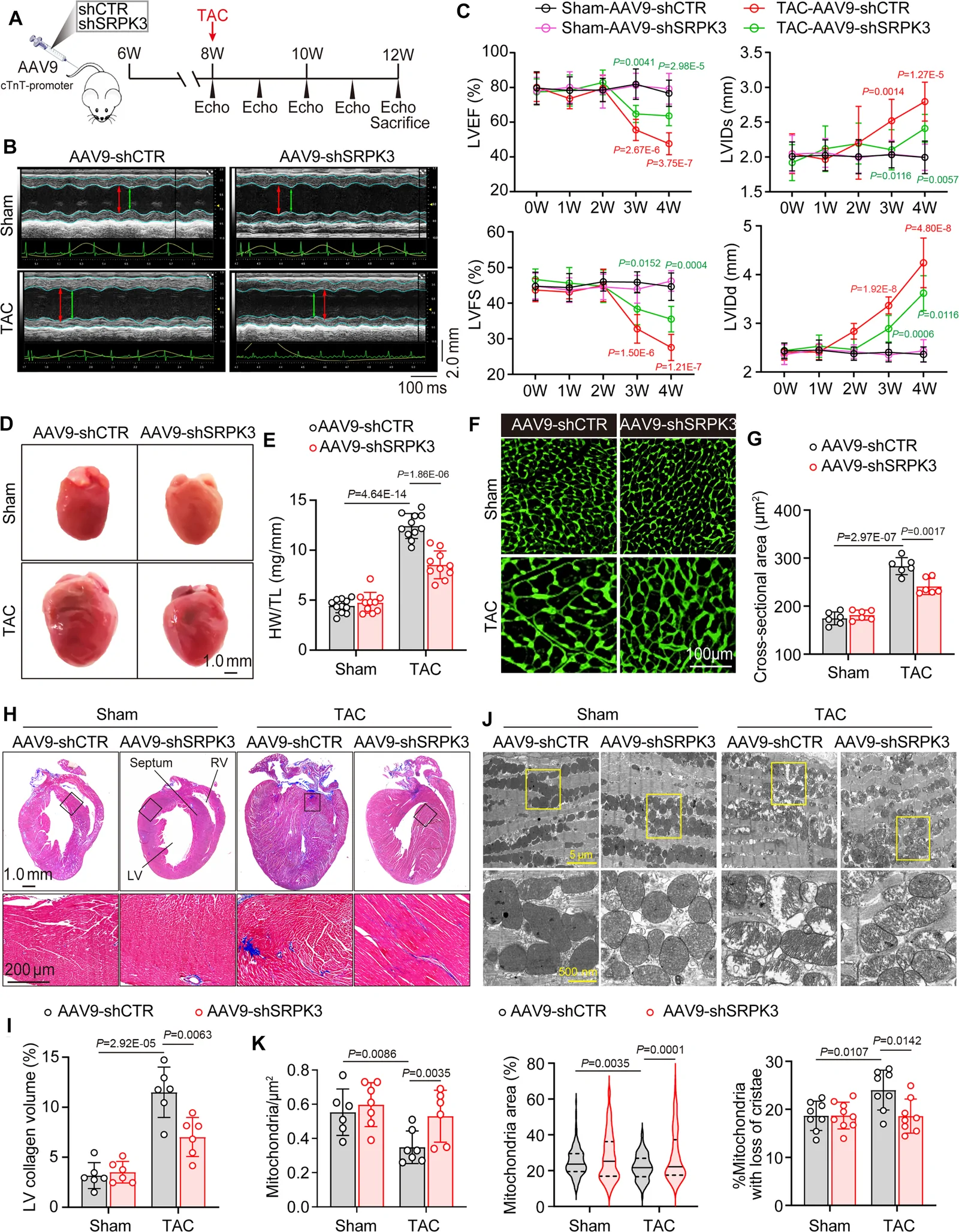
Silencing SRPK3 confers protection against pathological cardiac hypertrophy. (A) Schematical map of mice subjected to AAV9 administration after TAC surgery. (B) M model echocardiography of mice. (C) Echocardiography parameters including LV ejection fraction, LV fractional shortening, and LV end systolic/diastolic diameter were weekly recorded (*n* = 6 for each group). Red *P* value indicated TAC-vehicle versus sham-vehicle; green *P* value indicated TAC + AAV9-cTnT-shSRPK3 versus TAC + AAV9-cTnT-shCTR. (D) Representative anatomic images of mice. (E) Heart weight/tibia length (HW/TL) ratio of AAV9-shCTR (*n* = 11) and AAV9-shSRPK3 (*n* = 10) subjected to sham and AAV9-shCTR (*n* = 11) and AAV9-shSRPK3 (*n* = 10) subjected to TAC was calculated. (F) Representative WGA images and (G) quantification of CSA of myocytes of AAV9-shCTR (*n* = 6) and AAV9-shSRPK3 (*n* = 6) subjected to sham and AAV9-shCTR (*n* = 6) and AAV9-shSRPK3 (*n* = 6) subjected to TAC. (H) Representative images of Masson’s trichrome of hearts subjected to shSRPK3 or shCTR following sham or TAC surgery, and (I) LV collagen volume of AAV9-shCTR (*n* = 6) and AAV9-shSRPK3 (*n* = 6) subjected to sham and AAV9-shCTR (*n* = 6) and AAV9-shSRPK3 (*n* = 6) subjected to TAC was calculated. (J) Representative TEM images of LV myocardium. (K) Quantification of mitochondria-related parameters including mitochondria/μm^2^ of AAV9-shCTR (*n* = 6) and AAV9-shSRPK3 (*n* = 7) subjected to sham and AAV9-shCTR (*n* = 7) and AAV9-shSRPK3 (*n* = 6) subjected to TAC, mitochondria with loss of cristae of AAV9-shCTR (*n* = 8) and AAV9-shSRPK3 (*n* = 9) subjected to sham and AAV9-shCTR (*n* = 8) and AAV9-shSRPK3 (*n* = 8) subjected to TAC, and mitochondrial area from (J). Mitochondrial area refers to the ratio of mitochondrial area to image area (WT, *n* = 241; Srpk3 cTg, *n* = 263). *P* values were shown. One-way ANOVA with an LSD post hoc test was used for multi-group comparisons.

Having demonstrated the striking function of SRPK3 in postnatal cardiac growth, which prompts us to evaluate whether SRPK3 blockage confers cardioprotection in pathological hypertrophy. Here, we first administered AAV9-cTnT-shSRPK3 or AAV9-cTnT-shCTR to mice (2 weeks before TAC surgery by tail vein injection) and conducted weekly echocardiographic monitoring until sacrifice (Fig. 7A and Fig. S16A). Results indicated that Srpk3 knockdown in mice significantly alleviated cardiac pump dysfunction and cardiac output decline in TAC-treated mice, and these effects were not influenced by sex, according to our statistical analysis (Fig. 7B and C, Fig. S16B and C, and Table S6). In line with the attenuated hypertrophic phenotype in genetic Srpk3 deleted mice, animals that received shSRPK3 treatment exhibited decreased cardiac enlargement (Fig. 7D and E) and attenuated cellular CSA (Fig. 7F and G) at 4 weeks after TAC surgery. To identify the alteration of molecular mechanism, we probed the phosphorylation level of RPS3 and SRSF2 after AAV9-shSRPK3 injection and TAC surgery, and then the consequence of immunoblotting demonstrated that the depletion of SRPK3 can effectively suppress the phosphorylation induced by TAC (Fig. S16F and G). Morphologically, AAV9-cTnT-shSRPK3 injection improved the IVS thickening and slightly reduced collagen deposition in LV (Fig. 7H and I). Moreover, swollen mitochondria and apparent disruption of cristae were observed in pressure overloaded mice (Fig. 3H). Interestingly, these adverse alterations were abolished following AAV9-cTnT-shSRPK3 treatment (Fig. 7J and K). Immunoblotting also showed that knockdown of SRPK3 ameliorated the pathological cardiac hypertrophy and defective mitochondrial biogenesis (Fig. S16D and E). Collectively, these data demonstrated that silencing SRPK3 exerted cardioprotective benefits.

## Discussion

Defective mitochondria, characterized by extensive swollen fragments, cristae fracture, and mitochondrial DNA (mtDNA) loss [57], can contribute to the evolution of cardiac hypertrophy and HF. Indeed, emerging evidence has indicated that mitochondrial impairment can cause macrostructural remodeling [58,59]. Specifically, nuclear genome profoundly influences the mitochondrial proteome abundance through a process regulated by PGC1α as the key transcription factor of mitochondrial biogenesis and bioenergetics [60], despite that the 13 components were encoded by mtDNA. Nevertheless, the precise mechanisms governing nuclear genome-derived RNA metabolism remain to be determined, primarily due to the existence of numerous regulatory layers. In this context, our findings reveal that SRPK3 orchestrates its pro-hypertrophic effects through a combination of synergistic mechanisms within CMs. Initially, SRPK3 acts as a facilitator, promoting the assembly of phosphorylated SRSF2 in the nucleus, consequently leading to aberrant AS of RNA in CMs. This process aligns with the canonical modulation of SRPKs-SRSFs. In addition, our findings suggest a noncanonical function of SRPK3 in posttranscriptional repression preferentially promoting PGC1α mRNA decay through hyperphosphorylated RPS3^Ser149^, a ribosomal protein that acquires unconventional nuclear localization (Fig. 6M). Jointly, restoration of nuclear genome-derived RNA metabolic homeostasis by CRISPR-mediated ablation of SRPK3-SRSF2 binding and SRPK3-RPS3^Ser149^-PGC1α mRNA alleviated the impairment of mitochondrial biogenesis in hypertrophic human CMs. Therefore, based on our results, our findings propose an innovative approach for regulating RNA metabolism by targeting SRPK3 as a potential therapeutic approach for pathological cardiac hypertrophy.

The SRSFs are crucial splicing factors essential for AS, and the phosphorylation of SRSFs is tightly controlled by SRPKs, thus determining AS patterns of numerous mRNAs. In hypertrophic conditions, elevated SRPK3 in the nucleus is sufficient to recapitulate the adverse features of HCM and drives AS via SRSF2 phosphorylation, a mechanism analogous to the RNA chaperone function previously linked to SRPKs [45,61]. SRSF2, also known as pre-mRNA splicing factor SC35, acts as a prototypical SR protein that contains 2 RNA recognition motifs (RRMs) and a signature motif enriched in arginine/serine dipeptide repeats (RS domain) [62]. SRSF2 mutation has been broadly verified to be engaged in multiple steps of spliceosome assembly, thereby contributing to cancer pathogenesis [63]. Moreover, cardiac-specific ablation of SRSF2 has been shown to induce dilated cardiomyopathy (DCM) [25], and SRSF2 mutation is associated with the risk of atherosclerosis in patients [26]. Rather than merely altering SRSF2 expression levels, our investigation revealed the presence of hyperphosphorylated SRSF2 due to increased activity of SRPK3 in hypertrophic CMs. This phenomenon led to the aberrant splicing of mitochondrial genes. These findings offer mechanistic insights into the SRSF2-RNA splicing cascade, particularly concerning splicing factors implicated in disease pathogenesis. Most splicing alterations associated with SRSF2 mutations were driven by the dynamic cassette-exon splicing induced by SE [62,64,65], and the increase in SRPK3 expression triggered excessive SE splicing in CMs. CRISPR-editing SRSF2 dephosphorylation conferred cardioprotection against hypertrophic remodeling, by restoring the SRSF2-RNA splicing cascade.

The mitochondria occupied approximately 30% of the CM volume and provided more than 95% of the energy in CMs through OXPHOS [66]. Mitochondrial integrity ensures not only adequate ATP production but also intracellular calcium homeostasis, signal transduction, and cell survival [67]. Recent publications have highlighted the association between defective nucleus-encoded mitochondrial transcriptome and cardiac hypertrophy [68,69]. Our findings align with this, as we observed nuclear SRPK3-SRSF2 coupling mediating RNA splicing of genes related to mitochondrial function, thereby contributing to the development of hypertrophic cardiomyopathy. Importantly, our discovery revealed that the substantial increase in SRPK3 expression in CMs had negligible impact on mitophagy. This suggests that the decline in mitochondrial content mediated by elevated SRPK3 is primarily attributable to defective mitochondrial biogenesis rather than hyperactive mitophagy, despite the known association between mitophagy and cardiac hypertrophy.

RPS3, which is an RNA-binding protein, was initially identified as a component of the 40S ribosomal subunit in eukaryotes that physically help anchor mRNA to ribosomal subunits to maintain the activity of ribosomal helicase, thereby preventing mRNA dissociation after codon recognition initiation [70,71]. Nonribosomal free RPS3, rather than its ribosome-associated counterpart, has attracted substantial attention due to its involvement in DNA repair, apoptosis, and antibacterial innate immune responses [72,73]. In line with the nuclear RPS3 phosphorylation by protein phosphatase 2A and Akt (also known as protein kinase B (PKB)] [74,75], our results showed that SRPK3 physically interacted with and phosphorylated RPS3 at Ser^149^, thus promoting nuclear RPS3^Ser149^ enrichment in CMs under hypertrophic stimuli. RPS3-RNA binding triggers a poison 5′-splice site in pre-mRNA of SRSF7 and U2AF1 genes, leading to a frame shift and degradation by NMD [50]. Similarly, our finding indicated that RPS3^Ser149^ hyperphosphorylation induced by SRPK3 contributed to the degradation of PGC1α mRNA [76], thereby disrupting its stabilization and translation, as well as the degradation of PGC1α mRNA in CMs. However, the mechanism underlying the regulation of RPS3 on PGC1α RNA spliceosomes and the physical coupling of nuclear RPS3 and PGC1α mRNA remain to be clarified.

In summary, our findings demonstrate that SRPK3 is a potential therapeutic target for improving mitochondrial biogenesis and restoring impaired energy production in HCM and other hypertrophic cardiac phenotypes that feature mitochondrial dysfunction. Moreover, we identified a canonical SRPK3-SRSF2-mediated nuclear genome-derived RNA (that encodes mitochondrial protein) AS affecting genes related to mitochondrial function, along with a noncanonical role of SRPK3 involving binding of RPS3 Ser^149^ to destabilize and degrade PGC1α mRNA. These dual pathogenetic mechanisms offer a novel insight into molecular basis of concentric hypertrophy.

There are several limitations of the work conducted here. First, we applied the widely used TAC mouse model to mimic the pathogenesis of human cardiac hypertrophy. However, the trigger of human hypertrophy is typically genetic. Therefore, TAC-induced pressure overload hypertrophy is inconsistent with the human condition, making translation from this animal model to human disease unreliable. Besides, the majority of mitochondrial components in Srpk3 cTg exhibited an increase in SE splicing variants, with the exception of Bnip4 and Guf1, a finding that merits in-depth investigation. Although we reveal the pro-hypertrophic effects of SRPK3 under pathological stimulation, the link of SRPK3 and embryonic heart development is worth pursuing. Moreover, more detailed information of SRPK3-SRSF2 binding and RPS3-PGC1α mRNA awaits further investigation. A further limitation is that our manuscript insufficiently covers RBM20 (RNA-binding motif protein 20), a cardiac AS regulator [77]. RBM20 is linked to cardiomyopathy via its modulation of titin (TTN) splicing and is recognized as an HCM candidate gene [78]. Interestingly, an AS event within RBM20 itself produces an isoform that is up-regulated in HCM, further elevating RBM20 levels [79,80]. Also, in addition to nuclear genome-derived mitochondrial RNA metabolism that has been investigated in this study, mtDNA-derived mtRNA metabolism is poorly understood due to the complexity of mitochondrial inheritance. In this study, we did not obtain evidence that mtDNA-derived RNA metabolism is affected by SRPK3, and the role of SRPK3 in cytoplasm, especially in mitochondria, was undefined. Additionally, the argument that Ser^149^ mediates the RPS3–SRPK3 interaction would have been considerably reinforced by phosphatase and peptide competition assays of the anti-phosphorylated RPS Ser^149^ antibody. Furthermore, the added direct phosphosite confirmation by liquid chromatography–tandem mass spectrometry (LC-MS/MS) will convincingly solidify our conclusion, optimizing the logical coherence of our findings. Similarly, the detailed interplay between the SRSF2-mediated splicing pathway and the RPS3-dependent mitochondrial pathway remains to be fully elucidated and is acknowledged as a key limitation. Unfortunately, such validations were not feasible owing to constraints in our available equipment. Furthermore, illustrating genetic modification, such as SRSF2 phospho-deficient/phosphomimetic mutation and RPS3 phosphomimetic mutation in animal models, would provide more compelling evidence compared to mere cellular restoration. Additional research evaluating its efficacy in larger animal models, including pigs and nonhuman primates, is imperative for advancing toward clinical translation.

## Methods

Detailed methods are provided in the Supplementary Materials.

### Statistical analysis

Data are expressed as mean ± standard error of the mean (SEM). Normality was tested using Shapiro–Wilk normality test. Unpaired, 2-tailed Student’s *t* test was used to assess variables that were normally distributed. For variables without normality, the Mann–Whitney test was applied. For the comparison of more than 2 normally distributed groups, one-way analysis of variance (ANOVA) with least significant difference (LSD) post hoc correction was adopted. Kruskal–Wallis test with Dunn’s post hoc correction was performed on the comparison of more than 2 groups that were not normally distributed. Two-way ANOVA with Holm–Sidak’s post hoc correction was utilized to evaluate the comparison of more than 2 groups and 2 different factors. Categorial data were compared using Fisher’s exact test. Statistical tests were conducted in GraphPad Prism 9. Two-sided *P* values below 0.05 were considered statistically significant.

## Data Availability

Upon reasonable request, the corresponding author will make the data, methods, and materials available to any researcher to reproduce the results or replicate the procedure. The RNA-seq data and original sequence files that sustain the findings of this study are available on the Gene Expression Omnibus (GSE260494, GSE238123).

## Supplementary Materials

Supplementary 1 Materials and Methods Figs. S1 to S16 Tables S1 to S8 References [19,31,41,42,68,81–98] Excel file 1
