# Supplementary material for: Targeting SRPK3 Attenuates Cardiac Hypertrophy and Heart Failure by Improving Mitochondrial Bioenergetics through mRNA Splicing and Decay
Source: Research (Wash D C). 2026 Aug 24;9:1400. doi: 10.34133/research.1400 (PMC13500917; doi:10.34133/research.1400)
Supplement: Supplementary file 1 [file research.1400.f1.zip › Supplementary Materials(Clean Version with Author Info).docx]

**Supplementary Materials**

**Targeting SRPK3 attenuates cardiac hypertrophy and heart failure by improving mitochondrial bioenergetics through mRNA splicing and decay**

*Zhan* *et al.*

*Corresponding author:

Saiyang Xie. MD, PhD

Department of Cardiology, Renmin Hospital of Wuhan University, Jiefang Road 238, Wuhan 430060, P.R. China. Tel: +86 2788041911; Fax: +86 2788066234

Email: yangsai1995@whu.edu.cn

Wei Deng. MD, PhD

Department of Cardiology, Renmin Hospital of Wuhan University, Jiefang Road 238, Wuhan 430060, P.R. China. Tel: +86 2788041918; Fax: +86 2788066271

Email: [vivideng1982@whu.edu.cn](mailto:vivideng1982@whu.edu.cn)

**The PDF file includes:**

Materials and methods

Figure S1-16

Table S1-8

**Materials and methods**

***Ethics statement***

All the experimental animal procedures were approved by the Institutional Animal Care and Use Committee of Renmin Hospital, Wuhan University, and the mice were fed in specific pathogen-free facilities of Model Animal Center in Cardiovascular Institute of Wuhan University. All mouse experiments were conducted following the Guide for the Care and Use of Laboratory Animal guidelines (No. WDRM-20201006A).

***Patient samples***

Human samples were obtained from explanted hearts from 35 non-heart failure(HF) controls (mean age, 58.3±10.1 years; 19 males) and 47 HF patients (mean age, 53.7±9.6 years; 28 males) who had received heart transplants or endomyocardial biopsy at Cardiovascular Hospital affiliated with Renmin Hospital of Wuhan University (baseline characteristics in Table S1). Myocardial tissue was isolated from the patient’s left ventricle for research purposes. Disease etiology: pressure overload (~40%), ischemia (~30%), cardiomyopathy (20%), and other causes (10%). All patients had New York Heart Association (NYHA) class IV heart failure and were receiving guideline-directed medical therapy (GDMT) for heart failure. Tissue specimens were all obtained from the left ventricular myocardium. Sex information is provided in the corresponding table. The underlying tissue composition is exclusively cardiac muscle, without contamination from connective tissue. The samples for biochemical studies were placed in liquid nitrogen and then stored at -80 °C immediately after tissue procurement. Structural parameters, including left ventricular internal dimension at end-diastole and end-systole (LVIDD and LVISD), interventricular septal thickness (IVST), and posterior wall thicknesses (PWTs), were evaluated by echocardiography. LVH was defined by the partition values of (left ventricular mass) LVM body surface area (BSA) above 125 g/m^2^ for men and above 120 g/m^2^ for women as previously described(*81*). The LVM and BSA were calculated according to the following formula: LVM (g) = 0.8 × [1.04 × (LVIDd + IVST + PWT)^3^ − (LVIDd)^3^] + 0.6. BSA (m^2^) = 0.0061 × height (cm) + 0.0128 × weight (kg) −0.1529. Control human heart samples were taken from healthy donors who died unexpectedly and could not donate their hearts for noncardiac reasons. The patients and families of the donors provided informed consent. The protocol for this study complied with the Declaration of Helsinki and was approved by the review committee of the Renmin Hospital of Wuhan University (WDRY2018-K021).

***Plasmids and cloning***

Plasmids were purchased from MIAOLING Biology. Two plasmid backbones: pSpCas9(BB)-2A-Puro [Addgene #62988] (*82*) encoding tracrRNA (gRNA scaffold), Cas9, and puromycin resistance gene as well as Lenti CRISPR v2-Blast (Addgene #83480) encoding tracrRNA (gRNA scaffold), Cas9, and blasticidin resistance gene were used in the study. Three different sgRNAs recognizing either 5′ upstream region or 3′ downstream region of targeted genes (RPS3 Serine 149 locus and exons 32-42 of SRSF2) were designed using CRISPR Design Tool (http://crispr.mit.edu) software (Table S4). Plasmids were first digested with one of two restriction enzymes, BbsI (for digestion of pSpCas9(BB)-2A-Puro) or BsmBI (for digestion of Lenti CRISPRv2-Blast), dephosphorylated at 5′ extremities with FastAP alkaline phosphatase (Thermo Fisher Scientific) and subjected to electrophoretic separation. Proper fragments were isolated with a DNA gel recovery kit (Zymo Research) according to the manufacturer’s protocol. Pairs of DNA oligos encoding a single sgRNA were mixed, phosphorylated using T4 PNK (New England Biolabs), and subjected to duplex formation. Subsequently, digested plasmids and oligoduplexes were ligated using Quick Ligation kit (New England Biolabs) followed by the transformation of Stbl3 competent bacteria (Thermo Fisher Scientific) and further amplification and isolation of constructed plasmids using Plasmid MIDI AX kit (A&A Biotechnology), according to the manufacturer’s protocol.

Full-length and indicated fragments of RPS3 cDNA were cloned into a pCMV vector containing the N-terminal Flag epitope tag, and full-length and indicated fragments of SRPK3 cDNA were cloned into a pCMV vector containing the N-terminal HA epitope tag. HEK293T cells were transfected with indicated plasmids using Lipofectamine 6000 (Thermo Fisher Scientific). After 36 h, immunoprecipitation was performed using anti- DYKDDDDK-Tag mouse monoclonal antibody (5 μg antibody in 500 μl cell lysate, 8146, Cell Signaling Technology, AB_10950495) and anti-HA-Tag rabbit monoclonal antibody (3724S, Cell Signaling Technology, AB_1549585), followed by a Western blot to identify protein interactions.

***Cell culture and transfection***

293T cells were cultured in DMEM high glucose (4.5 g/l; Lonza) supplemented with 10% FBS (fetal bovine serum, Biowest) and 1% Penicillin-Streptomycin (Gibco) in standard conditions (37 °C, 5% CO2). hiPSCs were generated from peripheral blood mononuclear cells isolated from a healthy male donor upon obtaining informed consent. The reprogramming process was performed using CytoTune™-iPS 2.0 Sendai Reprogramming Kit (ThermoFisher Scientific)(*83*). Harvested cells were cultured on Geltrex (Thermo Fisher Scientific) coated plates in Essential 8 medium (Thermo Fisher Scientific) in standard conditions (37 °C, 5% CO2) and passaged with 0.5 mM EDTA when confluence reached 80%. Cells were transfected with plasmids expressing the base editing components using Lipofectamine 6000 (Thermo Fisher Scientific) according to the manufacturer’s instructions. Three days after the transfection, cells were harvested to assess the editing efficiency.

***Preparation of genic editing hiPSC ^RPS3 Ser149Ala^ and hiPSC ^SRSF2ΔRS^ cell lines***

hiPSCs were nucleofected with two plasmids encoding selected sgRNAs from Table S4: for RPS3 Serine 149 locus pSpCas9-2APuro-sgRNA1 and Lenti CRISPR v2-Blast-sgRNA2 (Table S4); for exon 32-42 of SRSF2 pSpCas9-2A-Puro-sgRNA1 and Lenti CRISPR v2-Blast-sgRNA1 (Table S4) using Human Stem Cell nucleofector kit 1 and A23 program on Amaxa Nucleofector 2b device(*84*). After antibiotic selection (blasticidin 20 μg/ml and puromycin 0.5 μg/ml) cells were seeded in low confluency (70 cells/cm^2^) to form single-cell clones. Selected colonies were mechanically dissociated , part of the collected cells was seeded for further culture and the rest subjected to DNA isolation using the Genomic Mini kit according to the manufacturer’s protocol. Deletion of exons 32-42 of SRSF2 and RPS3 Ser149 locus mutation in hiPSCs were evaluated with PCR using primers complementary to genomic regions flanking RPS3 Ser149 locus

(forward: 5′ TCCAAGAAGAGGAAGAACACAGA 3′,

reverse: 5′ CCTCGGAGTTTCCCAGACAC3′)

or exon 32-42 of SRSF2 (forward: 5′GACCGCTACACCAAGGAGTC 3′,

reverse: 5′ GCTTGCCGATACATCATTTTCT 3′)

and KAPA2G Fast Genotyping Mix. Amplified products were then separated in 2% agarose gel where bands shorter than in the control sample indicated clones with complete deletion of exon 32-42 of SRSF2 or mutation at the RPS3 Ser149 locus. These clones were selected for further culture.

To assess the off-target activity of sgRNAs used to generate SRSF2 exon 32-42-deficient and RPS3 Ser149 locus mutant hiPSCs, primers were designed to amplify 4 selected sites putatively recognized by sgRNAs according to CHOPCHOP software (<https://chopchop.cbu.uib.no/> ) (Table S5). DNA was isolated from control and RPS3 Ser149 locus mutant hiPSC as well as control and exon 32-42 of SRSF2-deficient hiPSC-derived cardiomyocytes (see detail description blow) using Genomic Mini kit according to the manufacturer’s protocol and then subjected to Surveyor nuclease assay as described above.

To precisely evaluate changes in off-target site 1 for RPS3 Serine 149 locus 5′ sgRNA1, a genomic region with potentially introduced nonspecific modifications was amplified using CloneAmp HiFi PCR Premix (Takara) and the primers indicated in Table S5, subjected to 2% agarose gel electrophoresis The target band was isolated from the gel using Zymoclean™ Gel DNA Recovery Kit (Zymo Research) and sent for Sanger sequencing (Genomed).

**Adeno-associated virus and viral delivery protocol**

8-week-old C57BL/6J mice were used for experimental pathological cardiac hypertrophy studies. 10 μL AAV (5×10^12^ vg/ml) containing Srpk3 shRNA (AAV9-cTnT-shSrpk3) was administered to C57BL/6J mice at the age of 6 weeks by tail vein injection for prevention. Mice were euthanized at 4 weeks after TAC for histological and molecular assessment. The control groups were all treated with negative control (AAV9-cTnT-shCTR) for the same indicated time. The shRNA sequences were: shCTR, 5′-AGTTCGCGAGTAGAAGTA-3′; shSrpk3, 5′-ATTGCCTTAGGATCCTTAAGCCA-3′.

***Differentiation of human iPSC into cardiomyocytes***

Human induced pluripotent stem cell-derived cardiomyocytes (hiPSC-CMs) were generated from hiPSCs purchased from Nuwacell by modulating the Wnt signaling pathway, as our previous methodological description(*85*). Briefly, hiPSCs were cultured on Matrigel-coated plates (Corning, 354230) with mTeSR-1 medium (Stem Cell Technologies, 05850). The mTeSR-1 medium was changed daily, and hiPSCs were subjected to mechanical passaging weekly with 1.0 mg/mL Dispase solution (Gibco, 17105-041). Induction of cardiac differentiation began when hiPSCs reached 90% cell confluence. During the first two days of induced differentiation, hiPSCs were treated with 10 μM CHIR99021 (a selective GSK-3β inhibitor) in RPMI 1640 medium supplemented with insulin-free B27, ascorbic acid, and penicillin to promote the differentiation of cells into mesoderm. On the third day of differentiation, basal culture medium was used to maintain the culture of hiPSCs. Then 5 μM IWR-1-Endo (a Wnt signaling pathway antagonist) was added to the basal culture medium for two days to promote the differentiation of mesodermal cells into cardiomyocytes. On the 7th day of differentiation, the medium was replaced with fresh basal RPMI medium. On the 8th day of differentiation, weak contractile beating of hiPSC-CMs was observed under the microscope. Two weeks after differentiation, hiPSC-CMs showed stable and significant contractile beat. We have verified that hiPSCs are differentiated into cardiomyocytes by immunofluorescence with anti-cTnT antibody (Proteintech, 26592-1-AP). Importantly, flow cytometry targeting anti-cTnT-FITC (Miltenyi, 130-129-271) was also performed to confirm the differentiation. In addition, the membrane currents and action potential were recorded by the whole-cell patch-clamp technique, by which representative plateau was recorded in ventricle-like CMs (VLCMs, approximately 81%), and diastolic depolarization in sinoatrial node-like pacemaker cells (SAMLPCs, approximately 3%).

***Simulated cell hypertrophy in vitro***

Angiotensin II (Ang II) causes a rapid induction of immediate-early genes and hypertrophy in the cardiac myocytes(*86*). Here, Ang II (1.0 μM)-treated hiPSC-CMs were utilized to model pathological cardiac hypertrophy in maximal degree. In addition, localized synthesis of insulin-like growth factors (IGFs) has been broadly implicated in skeletal muscle growth, hypertrophy and regeneration. Virally delivered IGF-1 genes induce local skeletal muscle hypertrophy and attenuate age-related skeletal muscle atrophy, restoring and improving muscle mass and strength in mice(*87*). Here, IGF-1 (10 nM)-treated hiPSC-CMs were used to model physiological myocardial hypertrophy.

***Transmission electron microscope (TEM)***

Transmission electron microscopy was used to observe changes in the morphology and structure of mitochondria, as previously described(*88*). After euthanasia, hearts were harvested from the indicated mice (n = 4–6 per group; biological replicates were derived from independent animals). For each heart, left ventricular wall specimens were assigned a random code before further processing to ensure unbiased handling.1.0 mm^3^ heart pieces cut from the left ventricular wall were fixed in 1.25% glutaraldehyde and 0.1 M sodium cacodylate buffer at 4°C overnight. Heart samples were then washed 3 times in 0.1 M sodium cacodylate, 10 minutes each time and then the samples were dehydrated, embedded, and randomly assigned to sectioning batches. Heart samples were cut into 90 nm thick sections after dehydration and embedding. Finally, a Hitachi HT7800 transmission electron microscope (Hitachi, Japan) was used to capture images of the mitochondria. For each biological replicate, at least 10–15 randomly selected fields per grid were imaged by an observer blinded to the experimental groups. Higher magnification images of transmission electron microscopy (TEM) revealed the presence of damaged mitochondria in hypertrophic hearts with an increase of disarrayed cristae and a reduced electron density in the matrix, which refer to "impaired mitochondria". To evaluate sarcomeric ultrastructure, additional images were acquired from the same embedded blocks at higher magnification. For each left ventricular specimen, at least 10–15 randomly selected fields per grid were captured using the same Hitachi HT7800 transmission electron microscope. Sarcomere organization was assessed by examining the alignment and regularity of Z‑lines and the integrity of M‑lines. Disrupted sarcomeres were defined as those showing fragmentation or broadening of Z‑lines, loss of M‑line electron density, or overt myofilament disarray. All sarcomeric assessments were also performed in a blinded manner by an observer unaware of the experimental groups. All mitochondrial injury assessments were performed in a blinded manner.

***ATP analysis***

The levels of cardiac ATP were measured by a commercial ATP assay kit (Beyotime, Shanghai, China), according to the manufacturer's instructions. Briefly, the indicated heart tissue was adequately lysed and centrifuged. Subsequently, the supernatants were mixed with ATP detection working solution. Using a microplate reader (BioTek Instruments, Inc., USA), the luminescence was recorded and the values of ATP were calculated. ATP levels were expressed as nmol/mg protein.

***Seahorse extracellular flux analyzer assays***

The oxygen consumption rate (OCR) of hiPSC-CMs subjected to SRPK3 overexpression or silencing were measured using a Seahorse XFe24 extracellular flux analyzer, as previously described(*89*). We performed glycolysis stress tests and energy substrate (glucose and palmitate) oxidation test together with mitochondrial stress test. Mito stress test drugs (Oligomycin-A and FCCP) working concentrations required for hiPSC-CMs were determined with dose-titration assays. Above 10^^5^ cells per well were cultured in V-7 Seahorse plates. hiPSC-CMs were incubated in glucose-free Seahorse assay media supplemented with 1mM pyruvate in the incubator without CO_2_ prior to the assay. Injectors were loaded to add 20mM glucose, 1.0μM Oligomycin, 1.0μM FCCP, 1.0μM Rotenone and 2.0μM Antimycin A during mitochondrial stress test. Exogenous glucose oxidation and others mitochondrial stress test parameters such as basal respiration, maximal respiration, spare capacity, ATP production, proton leak and non-mitochondrial respiration were calculated from the OCR measurements.

***Reverse transcription-polymerase chain reaction (RT-PCR)***

To minimize batch effects and ensure unbiased processing, samples were assigned random codes before RNA extraction and all subsequent steps. RNAs were extracted from indicated tissues using TRIzol reagent (Invitrogen), digested with Rnase-free DNase (Roche), and 1 μg of RNA was reverse-transcribed into cDNAs using the PrimeScript RT Reagent Kit (Takara) according to the manufacturer’s protocol. RT-PCR primers designed to amplify two or multiple isoforms with different sizes are shown in Table S6; the order of sample loading on gels was also randomized. Amplified PCR products were resolved by electrophoresis on 2% agarose gels in 1× TAE buffer at 100 V for 40 min, and visualized under UV light after ethidium bromide staining. PCR products were quantified using Image J software by an investigator blinded to the experimental groups. Splicing ratios were represented as PSI (Percent Spliced In) value, which representing the percentage of a gene’s mRNA transcripts that included a specific exon or splice site. For each biological replicate, PSI values were averaged from at least two independent RT-PCR runs.

***mtDNA copy number analysis***

mtDNA/nDNA ratio indicates the mitochondrial DNA copy number versus the nuclear DNA copy number, which is widely used to measure the mitochondrial abundance in tissues. Total DNA was extracted from 20mg left ventricular tissue using a commercial kit (QIAGEN, Cat. 69504) according to its instructions. To minimize batch effects, all samples were assigned random codes before DNA extraction, and the subsequent steps were performed in this randomized order. Mitochondrial DNA and nuclear DNA copy numbers were quantified by real-time polymerase chain reaction assays on the Roche LightCycler® 480 detection system. mtDNA-encoded MT-ND1 and nDNA-encoded MX1 were used as mtDNA and nuclear DNA markers, respectively. Primers specific for MT-ND1, forward: ctcttatccacgcttccgttacg; reverse: gatggtggtactcccgctgta. Primers specific for nDNA-encode Mx1, forward: gacataaggttagcagctaaaggatca; reverse: tctccgattaaccaggctagctat (*90*). Samples were loaded onto the PCR plate in a randomly allocated layout, and all measurements were performed in duplicate. Data analysis, including the calculation of mtDNA/nDNA ratios, was conducted by an investigator blinded to the group assignments.

***GST pull down assay***

The GST pull-down assay was used to detect the specific binding between RPS3 and SRPK3. Briefly, GST-tagged RPS3 (GST-RPS3) and His-tagged SRPK3 (His-SRPK3) proteins were expressed in Escherichia coli via transforming pGEX-4T-1-GST-RPS3 and pET24a-His-SRPK3 plasmids, respectively. Then, the Escherichia coli was collected, sonicated, and purified with His-Tag Purification Resin (Roche) to obtain purified His-SRPK3 protein. GST-RPS3 protein was purified and immobilized using BeyoGold™ GST-tag Purification Resin (P2250, Beyotime, China) according to the manufacturer’s instructions(*91*). As a negative control, GST alone was expressed from the pGEX-4T-1 empty vector in E. coli and purified and immobilized onto the same GST-tag Purification Resin in parallel to generate GST-bound beads. The GST-RPS3-bound beads and the GST-bound beads were washed with GST pull-down binding buffer and separately incubated with purified His-SRPK3. Finally, the beads were washed and analyzed by immunoblotting. To ensure reproducibility, all pull-down assays including both the GST-RPS3 group and the GST negative control group were performed with three independent biological replicates. The order of sample processing across different replicates and groups was randomized, and immunoblot image analysis was carried out by an investigator blinded to the experimental conditions.

***RNA-FISH***

The GFP-labeled *PPARGC1A* RNA probe was designed and synthesized by Creative Bioarray to target *PPARGC1A* mRNA. Briefly, the RNA probe targeting the end-to-head junction of PPARGC1A was hybridized to hiPSC-CMs. After fixing in 4% paraformaldehyde solution, samples were rinsed in 1×PBS, permeabilized in 1 × PBS with 0.5% Triton X-100 (10 min) and blocked in 3% BSA for 30 min at room temperature. Then hiPSC-CMs were incubated with anti-RPS3 primary antibody overnight. The cells were then applied with Alexa Fluor™ 568-conjugated secondary antibody (Thermo Fisher Scientific). Immediately after DAPI was added, images were taken with confocal microscope (Leica, TCS SP8). selected under low magnification before switching to higher magnification for image capture. Throughout the imaging session, the operator was unaware of the experimental conditions, and all subsequent image analysis was carried out in a blinded manner.

***Mitophagy assessment***

Keima is a fluorescent protein that emits different colored signals at acidic and neutral pH. Mito-Keima is a mitochondrially localized pH indicator protein described by Katayama et al.(*92*). We generated AAV9 carrying Mito-Keima. The method used to detect lysosomal delivery of Mito-Keima has been described(*92*). In brief, heart sections were subjected to confocal imaging using the Nikon A1RSI confocal microscope equipped with a 20× Plan Apo VC objective lens. Mito-Keima at neutral pH was detected by excitation with the 440 nm laser line and the acidic pH at 560 nm. Ratio (560/440) images of Mito-Keima were analyzed using Nikon NIS-elements software. AAV9 vectors were packaged with Mito-Keima, which was driven by the troponin T2 (Tnnt2) promoter. Doses of 5 × 10^10^-1 × 10^11^ particles of each vector were administered intravenously through the tail vein(*93*). Four weeks after virus administration in Srpk3 cKO or WT mice (n = 5 per group, biological replicates). Following this, they were euthanized. Transduction of AAV9-Mito-Keima was observed in heart tissue or isolated adult cardiomyocytes (AMCMs). For each sample, at least ten fields of view were randomly selected under low magnification before high-resolution acquisition. Throughout image acquisition and subsequent analysis, the investigator was blinded to the genotype and treatment group.

***Recombinant proteins and virus production***

Human SRPK3 (#Abnova, #P5605) and its substrate SRSF2(#ZY6321Hu011) were used. ATP was obtained from Sigma-Aldrich (#A7699), and a 10 mM stock was prepared in water just before use. SRPK3 was assayed in 70 mM HEPES, pH 7.4, 3 mM MnCl2, 3 mM MgCl2, 3 μM sodium orthovanadate, and 1.2 mM DTT (“kinase buffer”), as recommended by ProQinase. Kinase reactions were initiated with the addition of ATP and carried out at room temperature using a 10 μL kinase reaction volume in a 384-well AlphaPlate™. AlphaPlates are light gray plates that combine the advantages of white plates (high level of luminescence) and black plates (low levels of well-to-well cross-talk) for optimal luminescence assay performance. To terminate the kinase reaction at the indicated time points, Easylite-Kinase reagent was added in equal volume (10 μL/well) to the assay plate. The plate was covered, agitated for two minutes on a plate shaker (1100 rpm), and read on the VICTOR® Nivo™ (PerkinElmer) using standard luminescence settings. For physiological myocardial hypertrophy modeling, IGF-1 was obtained from MedChemExpress (MCE) (HY-P70783G).

In hiPSC-CMs, to overexpress SRPK3, cells were infected with adenovirus (Vigene Biosciences, China) expressing SRPK3(Ad-GFP-SRPK3) at a multiplicity of infection of 50 for 48 hours and control adenovirus(Ad-CTR) was used as a control. SRPK3 protein was detected by western blot to examine the overexpression efficiency of adenovirus-mediated overexpression. Besides, to knock down endogenous SRPK3, hiPSC-CMs were transfected with the short hairpin RNA (RiboBio, Guangzhou, China) against SRPK3 (shSRPK3, 50 nmol/L) using Lipo-6000 reagent (Beyotime, China) according to the manufacturer’s instructions, and scrambled control RNA (shCTR) was used as the negative control. The sequence of shSRPK3 was as follows: 5’-GAAAACUGCCUGUUUGUUU-3’, and shCTR was: 5’-UUCUCCGAACGUGUCACGUTT-3’. Total protein was extracted to determine the effectiveness of shRNA transfection by western blot.

***Coimmunoprecipitation***

For coimmunoprecipitation (Co-IP) assays, the primary cardiomyocytes were infected with indicated virus and HEK293T cells were co-transfected with indicated plasmids. Then cells were lysed in cold immunoprecipitation buffer as previously described(*81*). Heart tissues were fragmented with a Tissuelyser-24 at 60 Hz for 120 seconds at 4℃ after the addition of immunoprecipitation buffer. Samples were lysed on ice for 10 minutes and after centrifugation, the supernatant containing the total protein was collected. Protein concentrations in cells or myocardial tissues were measured using the BCA Protein Assay Kit (Thermo Fisher Scientific, USA). Sample lysates containing 1.0mg of total protein for immunoprecipitation (IP). Samples are incubated with specific antibodies and rotated at 4 °C for 4 hours. Subsequently, 20 μL protein A/G-agarose beads were added into the lysates and the mixture was rotated for 2 hours. The incubation order of samples across different groups was randomized. After being washed 3 times with immunoprecipitation buffer and boiled with 2×SDS loading buffer at 95 °C for 10 minutes, the eluted proteins were immunoblotted with the indicated primary antibodies. All immunoblot images were acquired and quantified by an investigator blinded to the experimental groups. Each Co-IP experiment was repeated in at least three independent biological replicates.

***Immunoprecipitation-mass spectrometry (IP-MS)***

Flag-SRPK3 or Flag-vector-transfected HEK293T cells were washed with phosphate-buffered saline (PBS) and lysed with immunoprecipitation lysis buffer containing 100 μM phenylmethylsulfonyl fluoride (PMSF; Beyotime). To ensure reproducibility, three independent biological replicates were prepared per condition, and the order of sample processing was randomized across replicates. The cell lysates were subjected to immunoprecipitation with 50 μl protein A-agarose beads and anti-Flag (1:50) antibody (Ab) or normal rabbit IgG (1 μg/ml) at 4 °C for 2–4 h. The candidate proteins interacting with SRPK3 were identified by mass spectrometry(supported by Shanghai Biotree technological Co., Ltd.). All samples were coded and analyzed in a blinded manner. Detailed information upon SRPK3 binding candidates was provided in Supplementary Excel file 1.

***Mice strain***

Adult wild-type C57BL/6 mice (8 to 10 weeks old, body weight (BW): 23.5–27.5 g) were purchased from the Institute of Laboratory Animal Science, Chinese Academy of Medical Sciences (Beijing, China). Allow experimental mice to adapt to the laboratory environment for 1 week. Moreover, mice with a conditional knockout allele of Srpk3 (Srpk3^fl/fl^) were constructed from Cyagen Biosciences Inc (Suzhou, China). To achieve this knockout, exons 6-8 were flanked by LoxP sites, and thus, two single guide RNAs targeting the exons were constructed. Purified Cas9 mRNA and two sgRNAs:

(sgRNA6: 5’-AAAGGCTACTTTTGAGGCCTACTT-3’

sgRNA10: 5’-AGGAGCACTTAGACTCCAGAAATG-3’) were then injected into zygotes, which were subsequently implanted into pseudopregnant mice. From 102 transferred zygotes, we identified one founder (#11) that possessed floxed 6-8 exons within the same allele. Successful generation of this conditional knockout allele was validated by touchdown PCR with the KOD-FX enzyme (#KFX-101, TOYOBO, Osaka, Japan) using the Srpk3-specific primers presented at fig.6B and 8B Mice carrying the *Myh6*-MerCreMer construct (A1cf^Tg(Myh6-cre/Esr1*)1Jmk^, strain #005657) were obtained from The Jackson Laboratory; these mice were used in our previous studies(*94*). Cardiomyocyte-specific Srpk3 knockout (Srpk3 cKO) mice were generated by mating Srpk3^fl/fl^ mice with *Myh6*-MerCreMer mice.

Mice with conditional transgenic expression of Srpk3 (Srpk3^Tg/+^) were generated through the utilization of a Rosa26 knock-in approach(*95*) and were backcrossed with C57BL/6 mice for at least seven generations. Srpk3^Tg/+^ mice were crossed with *Myh6*-MerCreMer mice to generate Srpk3^Tg/Tg^*-Myh6*-MerCreMer mice.

***Mouse surgery and functional assessment***

Work overload induced by transverse aortic constriction (TAC) is a well-studied mouse model of pathological cardiac hypertrophy. Briefly, these mice were anesthetized by intraperitoneal injections of 3% pentobarbital sodium, and the adequacy of anaesthesia was assessed by the pedal withdrawal reflex, breathing, and response to operation. The left chest was exposed by performing a Mini sternotomy, and the thoracic aorta was identified at the second intercostal space. Transverse aortic constriction was performed with a 27-G needle using a 7-0 silk suture. After that, the needle was removed, and the thoracic cavity was closed. During the surgery, the body temperature was maintained by a warming pad. Temgesic (0.1 mg/kg/day) was used to relieve postoperative pain. The adequacy of ligation was confirmed by validating increased blood flow velocity and pressure gradient of left and right common carotid arteries by using Doppler echocardiography. The mice in the sham groups underwent a similar procedure without ligation. Mice were sacrificed by cervical dislocation at the end of these experiments.

Echocardiography was performed following TAC for 4 weeks or swimming exercise. As our previous study(*81*), mice were anesthetized (2% isoflurane) and echocardiography was analyzed with a 10-MHz linear array ultrasound transducer equipped with a 30-MHz probe (Vevo 3100 system Visual Sonics). Some parameters were measured under the long-axis M-mode. Notably, the fractional shortening (FS) was calculated by the formula: LVFS = (LVIDd-LVIDs) × 100/LVIDd. The LV ejection fraction (LVEF) was also evaluated using the Teichholtz formula: LVEF = ([100-Y] × 0.15) + Y; Y = (LVIDd^2^-LVIDs^2^) × 100/LVIDd^2^(*81*).

***Single-cell capture and library preparation***

All single-cell capture and library preparation were performed at the Frasergen Technology co.ltd.(Wuhan, China). The LV tissue of the mice was washed with 4°C normal saline solution containing 3% FBS (Thermo Fisher Scientific, Inc.) to remove attached blood clots. The tissue was transferred into a sterile RNase-free culture dish containing an appropriate amount of calcium-free and magnesium-free 1 × PBS on ice. And then, it was cut into pieces as small as possible and dissociated into individual cells according to the protocol of the 10 × Genomics platform. After centrifuged and the supernatant was removed, the pelleted cells were suspended in red blood cell lysis buffer (Solarbio, #R1010) and incubated on ice for 2 min to lyse red blood cells. After lysis, cells were centrifuged, washed, and resuspended in PBS with 0.04% BSA. Then cells were loaded into a 10x Genomics microfluidics chip and encapsulated with barcoded oligo-dT-containing gel beads using the 10x Genomics Chromium controller according to the manufacturer’s instructions. Single-cell libraries were then constructed according to the manufacturer’s instructions. Single-cell suspensions were loaded to 10x Chromium to capture 5000 single cells according to the manufacturer’s instructions of 10X Genomics Chromium Single-Cell 3’kit (V3). During the entire procedure, sample identity was concealed, and operators were blinded to group allocation. The following cDNA amplification and library construction steps were performed according to the standard protocol. Libraries were sequenced on an Illumina NovaSeq 6000 sequencing system (paired-end multiplexing run, 150bp×2) at a minimum depth of 20,000 reads per cell. Data analysis was also conducted with the analyst blinded to the experimental groups.

***Analysis of scRNA and snRNA-Seq data.***

Gene expression matrices were generated using the CellRanger software (10x Genomics) (https://10xgenomics.com/). Sample data was aggregated using CellRanger software and raw data was processed further in R (version 3.4.4 and 4.1.1). All analyses were conducted on samples from independent biological replicates (n = 3 per group), and processing order was randomized across groups. The following quality control steps were performed: (i) genes expressed by less than 10 cells or with a mean expression level below 0.002 were not considered; (ii) cells that expressed fewer than 500 genes (low quality), and doublets were excluded from further analysis using the doubletFinder function from doubletFinder_v3 package; (iii) cells in which over 25% of unique molecular identifiers (UMIs) were derived from the mitochondrial genome were removed. The data were normalized using the Normalize Data function as implemented in the Seurat package version 3.0 and 4.0. Subsequently, principal component analysis (PCA) reduction was performed on the scaled data and the first 15 principal components were used for graph-based clustering with a resolution of 1.2 Then Clusters were visualized by performing t-distributed stochastic neighbor embedding (t-SNE) using the original Louvain graph-clustering method. Throughout all computational steps, the analyst was blinded to the experimental groups. The conserved (marker) genes for each cluster were identified by using the function Find All Markers in Seurat. Cell clusters were annotated based on the high expression of known marker genes. Heatmaps were generated using Complex Heatmap (v. 4.1) with z-score transformed normalized gene expression values. Differential Gene Analysis and Functional Annotation: we identified differentially expressed genes in Srpk3 cTg mice and WT mice left ventricle for each type of cell, with a UMI count of each single cell by using the Seurat function Find Markers. Genes with a threshold of absolute fold change >1 and a false discovery rate (FDR) adjusted p-value <0.05 were considered as differentially expressed genes (DEGs). Gene ontology analysis was performed on DEGs by using an R package Cluster Profiler. The junction score for cell-cell and cell–extracellular matrix (ECM) was calculated by using the genes’ mean expression value for each cell type. The mean value was obtained by using the function Average Expression in Seurat. All differential expression and enrichment analyses were performed in a blinded fashion.

***RNA sequencing and analysis***

Total RNA was isolated from cardiac tissue of transgenic mice or genetically edited cellsby TRIzol Reagent (Invitrogen, 15596-026) and RNeasy mini kits (QIAGEN). To minimize batch effects, samples were randomly assigned a processing order before RNA extraction. RNA quality was assessed with a Agilent 2100 Bioanalyzer, showing high RNA purity and integrity. RNA-seq libraries were prepared according to NEBNext Ultra II Directional RNA Library Prep Kit (New England Biolabs, Massachusetts, USA) and sequenced using an Illumina HiSeq 2500 System (single-read 60bp). Library preparation and sequencing were performed by the CNIC’s Genomics Unit. FastQ sequencing files were then processed and analyzed with the web tool RNA-seq. Differentially expressed genes between groups were determined using moderated t-test from the limma package and adjusted by Benjamini-Hochberg (P<0.1). Gene set enrichment analysis was performed against KEGG pathway gene sets. Only significant enrichments with a Benjamini Hochberg-adjusted P value of less than 0.01 were considered. For heatmaps, leading edge gene sets from selected enriched pathways were extracted, annotated with their respective TPM values, which were then converted into raw Z-scores and imported into GraphPad Prism 9.0 for heatmap generation. All differential expression and enrichment analyses were carried out by an analyst blinded to group assignments.

***Differential splicing and expression analysis***

Clean reads were inspected with FastQC v0.11.3. Read mapping was performed with STAR 2.7.1a; the STAR 2-pass function was used to improve the detection of splicing junctions(*96*). The genome annotation file was downloaded from GENCODE vM22(*97*) under the mouse genome sequence GRCm38.p6. Samples from each group were processed in a randomized order to minimize batch effects, and all subsequent analyses were performed by an investigator blinded to group allocation. Exon-centric splicing events between two conditions were identified and quantified with the rMATS package (v.4.0.2) (*98*)using the default parameters. The following pairs of conditions were examined: Srpk3 cTg mice versus litter WT mice. The BAM files from STAR were used as the input for rMATS. Five major categories of AS events, including exon skipping, 5' alternative splicing, 3' alternative splicing, mutual exon exclusion, and intron retention, were detected from the RNA-Seq mapping results in rMATS software. The exon inclusion level was calculated from the count of reads specific to the exon inclusion isoform and the exon skipping isoform. Differential splicing events were considered significant with the criteria of a q(FDR)<0.1 and an absolute |ΔPSI| > 0.1. Finally, all genes with differential splicing events were used for cellular localization analysis based on the COMPARTMENTS database(*99, 100*).

***RIP sequencing***

RNA immunoprecipitation (RIP) was performed using hiPSC-CMs from at least three independent biological replicates per condition. To minimize batch effects, samples were assigned random codes and processed in a randomized order throughout the entire procedure. After the trypsinization, hiPSC-CMs were lysed in buffer containing 100 mM KCl, 10 mM HEPES (pH = 7.0), 0.5% Triton X-100, 5 mM MgCl_2_, 1 mM DTT, 0.5% NP-40, RNase inhibitor (100 U/ml) (Invitrogen), and EDTA-free proteinase inhibitor cocktail (Roche). Then, the cell lysate was then passed through a 27.5-gauge needle five times to promote nuclear lysis, followed by brief sonication using a Bioruptor 200 (Diagenode). After incubation on ice for 20 min, the nuclear lysate was added with 50 μl beads for pre-cleared incubation at 4 °C for 1 h. For each reaction, 5 μg RPS3 antibody (or IgG for controls) was incubated with Dynabeads Protein G (Invitrogen) in 1 ml NT2 buffer. After agitating at 4 °C for 4 h, control IgG and antibody-coated beads were incubated with hiPSC-CMs extracts and were agitated gently overnight at 4 °C. The next day, the bead complexes containing antibodies, target proteins, and RNA were washed for 20 min at 4 °C and repeated three times, as previous description(*101*).

Protein-bound mRNAs were then extracted using an RNA extraction kit (ZYMO research). The purity and integrity of eluted RNA were assessed using an Agilent Bioanalyzer. One to two micrograms of cDNAs were synthesized from the extracted RNA using the Clontech SMARTer cDNA kit (Clontech Laboratories, CA USA, catalog# 634938), and adapters were removed through digestion with RsaI. According to the manufacturer’s protocol, the cDNAs were fragmented using an ultrasonicator and profiled using an Agilent 2100 Bioanalyzer, followed by subjected to Illumina library preparation using NEBNext reagents (New England Biolabs, Ipswich, MA USA, catalog# E6040). Thereafter, the size distribution, quantity, and quality of the Illumina libraries were measured by the Agilent Bioanalyzer. The libraries were submitted for Illumina HiSeq2000 sequencing (Otogenetics, Norcross, GA). rRNA and globin RNA were removal during DNA library preparation. Paired-end 100 nucleotide (nt) reads were generated, and the data quality was checked using FASTQC (Babraham Institute, Cambridge, UK). The raw data were then subjected to data analysis using Tophat2 and Cufflinks. Two biological replicates were analyzed for each sample. All sequencing data were analyzed by an investigator blinded to the experimental groups.

***Combined RNA-Seq and RIP-seq data analysis***

To minimize potential batch effects, the order of sample processing and library preparation was randomized before sequencing. All subsequent bioinformatic analyses were conducted by an investigator blinded to the experimental groups. All sequencing data from the Illumina HiSeq X-ten platform or Illumina NovaSeq 6000 (Berry Genomics) were trimmed with Cutadapt (v2.3) to remove adapter sequences and low-quality reads. The quality control of RNA-Seq and RIP-seq data was performed

using FastQC (v0.11.8) and MultiQC (v1.11) and all trimmed data passed this step. The trimmed sequencing reads were further mapped to the mm10 reference genome using STAR (v 2.7.8a) with the “–out Filter Intron Motifs Remove Noncanonical Unannotated” parameters. The bigWig file for each sample was normalized according to the sequencing depth using the BEDtools (v2.29.2) genomecov function, for visualization in IGV (v2.11.0). The expression level of each transcript was quantified as transcript per million (TPM) using Salmon (v0.8.2), including RefSeq annotated RNA transcripts and transposon sequences downloaded from Repbase (v25.06). We used the pipeline, PipeRNAseq (<https://github.com/sunyumail93/PipeRNAseq> ), in our data processing to enhance the automation and reproducibility of the above steps. Salmon output files were further imported in R 3.5.0 using the tximport (v1.20.0) package for downstream analysis. Differential expression analysis was performed using DESeq2 (v1.32.0) package with adjusted p value < 0.05. Functional enrichment analysis was performed using clusterProfiler (v4.0.0) package, and visualized using “enrichGO”,“barplot” and “dotplot” functions. We focused on Gene Ontology terms including Molecular Function, Cellular Component, and Biological Process. We also summarized the downstream analysis into a wrapper R package, easyRNAseqDE (<https://github.com/sunyumail93/easyRNAseqDE> ). To specifically visualize RNA-Seq and RIP-seq data mapped to each transposon family, paired-ends reads were mapped directly to transposon consensus sequences using bowtie2 (v2.3.5.1). The un-mapped reads were filtered out using Samtools (v1.11), and mapped fragments were inferred from the mapped read pairs. We finally plotted the mapped fragments on each transposon consensus sequence and visualized the signals using R (v3.5.0).

***PPARGC1A mRNA decay assay***

An RNA decay assay was performed as previously described with some modifications(*102*). Cardiomyocytes of WT and RPS3 Ser149 mutant were seeded at equal density and cultured under identical conditions. Three independent biological replicates were performed per experimental group. Prior to treatment, the order of sample processing was randomized across all time points and groups. In brief, cardiomyocytes of WT and RPS3 Ser149 mutant were treated with actinomycin D (Sigma, A9415), and then samples were collected at 0 hour, 2 hours, 4 hours, and 8 hours after actinomycin D treatment. Total RNA was extracted and purified using the same method as that used for RIP-seq, and cDNA was prepared with a Super Script III First-Strand Synthesis System using random hexamer oligonucleotide primers. PPARGC1A RNA was detected by real-time PCR analysis, and the expression was normalized to the expression of the control gene 18S rRNA. All qPCR measurements were performed in duplicate, and data analysis was carried out by an investigator blinded to the time-point and group identities.

***Non-cardiomyocytes and cardiomyocyte isolation***

The methods for isolation of adult mouse cardiomyocytes have been described in our previous study(*85*). Briefly, adult mice were euthanized and their hearts were obtained by aseptic surgery. The harvested hearts were pre-cooled in Ca^2+^-free Tyrode solution at 4°C, after which the hearts were mounted on a Langendorff detached cardiac perfusion system. Then, hearts were immersed and perfused with calcium-free perfusion buffer for 3 mins, followed by 1.5g/L type II collagenase digestion for 10 mins. The heart was chopped up and digested into very small pieces of 1∼2 mm^3^ in size to separate individual cardiomyocytes. The morphology of AMCMs was observed using an inverted microscope. The cardiomyocytes were stained with a-actinin by immunofluorescence assay to confirm the purity.

After cells isolation from adult mouse hearts, fluorescence-activated cell sorting (FACS) was used to accurately separate cardiomyocytes, endothelial cells and cardiac fibroblasts. Briefly, single-cell suspension was resuspended in FACS buffer (2% FBS and 2 mM EDTA in PBS). To isolate cardiomyocytes, cardiac fibroblasts, and endothelial cells, cells were incubated at 4℃ for 30 minutes in the dark with the following antibodies: FITC-conjugated cTnT (Miltenyi, 130-129-271), Alexa Fluor® 647-conjugated Vimentin (V9) (SantaCruz, sc-6260AF647), PE-conjugated CD31 (BD Pharmingen, 555027). In addition, propidium iodide (PI) (20 μg/mL, Servicebio, #GA1174) was added to screen out viable cells and only viable cells were sorted. Subsequently, the stained cells were then washed twice in FACS buffer and were sorted in a fluorescence-activated cell sorting (FACS) cell sorter (BD Biosciences, FACS AriaIII).

***Tissue collection and immunostaining***

Hearts were harvested and fixed with 10% neutral formalin buffer overnight, as previously described(*85*). Before processing, all samples were assigned random codes, and the subsequent steps were performed in this randomized order. After dehydration and paraffin embedding, the samples were cut transversely into 5.0μm thick slices on a sliding section machine (Leica, Nussloch, Germany). We observed the general morphology of the heart under a light microscope and photographed the sections. For Masson’s trichrome staining, paraffin sections needed to be treated with xylene to remove the paraffin wax, sections were then rehydrated in graded ethanol series . Afterwards, the sections were stained with Masson’s trichrome staining using a Masson Trichrome kit (Servicebio, G1006, China) according to the manufacturer's instructions. For wheat germ agglutinin (WGA) staining, paraffin sections were stained with WGA to evaluate the cross-sectional area size of cardiomyocytes. Images were captured from at least 15 randomly selected fields per section, and CSA was quantified using ImageJ software by an observer blinded to the experimental groups. The membranes were stained with FITC-conjugated WGA, exhibiting green fluorescence. The cross-sectional area size of cardiomyocytes was measured by ImageJ software. For tissue immunofluorescence assays, sections was used to detect SRPK3 expression levels, SRSF2 localization, and α-Actinin in cardiomyocytes. Sections were incubated with primary antibodies (1: 100; diluted in PBS containing 1% goat serum) at 4°C overnight. The next morning, sections were incubated with secondary antibodies (1: 200; diluted in PBS) at 37°C for 60 minutes. Finally, DAPI was added dropwise to the sections. Heart sections were photographed with a confocal microscope (TCS SP5 Leica, Leica Microsystems GmbH, Wetzlar, Germany) equipped with an oil immersion objective. Images were acquired with a fluorescence microscope (Apotome, Zeiss, Oberkochen, Germany) and quantified with ImageJ software. All image analyses were performed in a blinded fashion. For immunofluorescence staining of hiPSC-CMs, WT and transfected cells were seeded onto gelatin-coated glass coverslips placed in 24-well plates at equal density and cultured under identical conditions. Following the designated experimental treatments, cells were washed with PBS and fixed with 4% paraformaldehyde for 15 minutes at room temperature. After permeabilization with 0.2% Triton X-100 in PBS for 10 minutes, cells were blocked with 5% goat serum in PBS for 1 hour at room temperature. Subsequently, cells were incubated with primary antibodies (1:100; diluted in PBS containing 1% goat serum) overnight at 4°C. The next day, after washing with PBS, cells were incubated with fluorescence-conjugated secondary antibodies (1:200; diluted in PBS) for 1 hour at room temperature in the dark. Nuclei were counterstained with DAPI, and coverslips were mounted onto glass slides with anti-fade mounting medium. Fluorescence images were captured using a fluorescence microscope (Apotome, Zeiss, Oberkochen, Germany) or a confocal microscope, with at least 15 randomly selected fields imaged per group. All image quantifications (e.g., fluorescence intensity, subcellular localization analysis) were conducted with ImageJ software by an investigator blinded to the group assignment. To ensure reproducibility, three independent experiments were performed, and the order of sample processing across different groups was randomized.

***Immunohistochemical (IHC) analysis***

To detect the expression of SRPK3 in the heart, human heart sections were used for immunohistochemical staining. The main steps of the experiment were described in our previous study(*81*). Human hearts were fixed in 4% paraformaldehyde overnight. After dehydration and paraffin embedding, the tissues were cut into 5.0μm thick sections on a sliding microtome. After dewaxing and hydration, sections were subjected to antigen retrieval by microwave heating (10-15 min) in citrate buffer. Sections were incubated with 3% hydrogen peroxide for 15 minutes to suppress endogenous peroxidase and blocked with 5% Goat Serum for an hour at room temperature to non-specific background staining. Sections were then incubated with SRPK3 primary antibodies in PBS (1:100) at 4°C overnight. Next, Sections were further processed with anti-rabbit/mouse GTVision1M+Detection System/Mo&Rb (Gene Technology, China) at 37 °C. One hour later, the samples were visualized by using diaminobenzidine (Gene Technology, China). Images of at least 10 randomly selected fields per section were captured with an Olympus BX41 microscope (Olympus, Milan, Italy) equipped with an Olympus DP50 camera at a magnification of 20x or 40x. Throughout image acquisition and analysis, all investigators were blinded to the group allocation. Quantitative analysis was performed independently by two blinded observers.

***Quantitative reverse transcription PCR***

Tissue RNA was extracted from mouse heart, liver, kidney, fat samples and so on. Specially, mice heart samples were acquired from left ventricular tissue, liver samples were obtained from the right lobe of the liver, and fat samples were collected from epididymal white adipose tissue. And mice kidney samples were obtained from the renal parenchyma. Brain samples were acquired from the cerebral cortex, skeletal muscle samples were collected from the gastrocnemius muscle, lung samples were obtained from the right lung lobe, and spleen samples were collected from the whole spleen. To minimize batch effects and ensure unbiased processing, all samples were assigned random codes before RNA extraction, and the subsequent steps were carried out in this randomized order. Cellular RNA was obtained from cardiomyocytes, fibroblasts, and endothelial cells isolated from mouse hearts, with at least three independent preparations per cell type. Both tissue RNA and cellular RNA were extracted using TRIzol reagent (Invitrogen, 15596-026) according to manufacturer’s protocol. cDNA was synthesized using the Transcriptor First Strand cDNA Synthesis Kit (Roche Diagnostics, Basel, Switzerland, 04896866001). The cDNA fragments were amplified on the Roche LightCycler® 480 detection system with FastStart Essential DNA Green Master (Roche, 04707516001). All primers used in this experiment were described in Table S7. GAPDH was used as an endogenous reference gene in this experiment. Throughout qPCR setup and data analysis, the operator was blinded to the group assignments.

***Fraction extraction***

Nuclear and cytoplasmic fractions of MEF cells were separated and extracted by NE-PER™ Nuclear and Cytoplasmic Extraction Reagents (Thermo Scientific, #78833) according to the manufacturer’s protocol. The protein level of β-Tubulin (cytoplasmic) and Lamin B1 (nuclear) in the cytoplasmic and nuclear fraction was checked to estimate separation efficiency.

***Cellular immunofluorescence assay***

Immunofluorescence staining was applied to confirm the co-localization of SRPK3 with phosphorylated RPS3 and SRSF2 in the nucleus and to detect the expression level of SRPK3 in cardiomyocytes of different treatment groups. All experiments were carried out on at least three independent biological replicates per condition. Prior to staining, coverslips were randomly assigned a code to conceal group identity, and all subsequent steps were performed in this randomized order. Briefly, cells on coverslips were fixed with 4% paraformaldehyde for 15 minutes at room temperature. Cells were permeabilized in 0.2% Triton X-100 for 10 minutes and then blocked with 8% Goat Serum for an hour at room temperature. Cells on coverslips were incubated with primary antibodies overnight at 4℃ and then treated with secondary antibodies for 60 min at 37℃. At last, DAPI was added to the coverslips to stain the nuclei. After washing, Fluorescence images were captured using a confocal microscope with an oil immersion objective; for each coverslip, 10–15 fields of view were randomly selected under low magnification before high‑resolution capture.. Throughout the imaging session, the operator was unaware of the experimental groups. ImageJ software was used for the quantitative analysis of the images in a blinded manner.

***Western blot analysis***

Mice heart samples were acquired from left ventricular tissue, liver samples were obtained from the right lobe of the liver, and fat samples were collected from epididymal white adipose tissue. And mice kidney samples were obtained from the renal parenchyma. Brain samples were acquired from the cerebral cortex, skeletal muscle samples were collected from the gastrocnemius muscle, lung samples were obtained from the right lung lobe, and spleen samples were collected from the whole spleen. The tissues samples were quickly harvested, rinsed in saline and then dropped into liquid nitrogen for immediate freezing. To minimize batch effects, all samples were assigned random codes before homogenization, and subsequent processing was performed in this randomized order. Homogenized tissues after grinding or cell were lysed in 1x RIPA lysis buffer (Invitrogen, Carlsbad, CA, USA) containing protease inhibitors. The tissue lysate or cell lysate is centrifuged and the supernatant is regarded as the total protein sample. Notably, nuclear protein fractions and mitochondrial protein fractions were separated by a commercial kit as previously described(*81*). Nuclear and Cytoplasmic Protein Extraction Kits (Beyotime, P0027) were used to extract nuclear and cytoplasmic proteins from fresh myocardial tissues and cells for subsequent experiments. The Bicinchoninic Acid Protein Assay kit (Thermo Fisher Scientific, Inc., Waltham, MA, United States) was performed to determine protein concentrations. Total protein was separated by 10% SDS-PAGE and subsequently transferred to a PVDF membranes (Millipore, Billerica, MA, USA). The PVDF membranes were incubated with primary antibodies at 4℃ overnight. The primary antibodies that specifically recognized the target proteins were listed in Table S7. The next morning, the membranes were incubated for one hour with secondary antibodies which were used to display primary antibodies bound to the target protein. After binding to enhanced chemiluminescence (ECL) substrates, the membranes were visualized on Bio-Rad ChemiDoc™ XRS+. Densitometric analysis was performed using Image Lab software by an investigator blinded to the experimental groups. Nuclear protein expression was normalized to Lamin B1, and cytoplasmic protein expression was normalized to β‑Tubulin. All immunoblotting experiments were repeated in at least three independent biological replicates.

***Statistics***

Data are presented as mean ± standard error of the mean (SEM). All animal assignments to experimental groups and the order of sample processing were randomized before the study began. Investigators performing data collection and statistical analysis were blinded to group allocations. Normal distribution was tested using Shapiro-Wilk normality test. Unpaired, two-tailed Student’s t test was used for variables that were normally distributed. For variables that were not normally distributed, the Mann-Whitney test was applied. For the comparison of more than two groups that were normally distributed, one-way ANOVA with LSD post-hoc correction was used. Kruskal-Wallis test with Dunn’s post-hoc correction was applied for the comparison of more than two groups that were not normally distributed. Two-way ANOVA with Holm-Sidak’s post-hoc correction was used for the comparison of more than two groups and two different factors. Categorial data were analyzed using Fisher’s exact test. Statistical tests were performed in GraphPad Prism 9. Two-sided p-values below 0.05 were considered statistically significant.

**Supplementary Figures**

**Fig. S1. Cardiac RNA splicing kinase SRPK3 was identified after work overload.** **(A)** Schematical map of pressure overload model by transverse aortic constriction (TAC), and pressure gradient of left and right common carotid arteries was assessed by using doppler echocardiogram.

**(B)** Gene ontology terms associated with the RNA metabolic genes differentially expressed in mouse hearts subjected to TAC compared to mice with Sham (GSE260492).

**(C)** Differential expression of RNA metabolic genes in mouse hearts with or without TAC.

**(D)** SRPK3 mRNA expression in several organ of human, as sourced from Fagerberg L et al. publication(*103*).

**(E)** Basic mRNA expression of Srpk3 in mouse brain, epididymal adipose tissue (eWAT), lungs, kidneys, skeletal muscles, liver, heart, spleen, normalized to Srpk3 mRNA in brain tissue(n=4 for each group).

**(F-G)** Protein levels and quantitative analysis of Srpk3 inside multiple organs(n=6 for each group), normalized to Srpk3 proteins in the brain.

Unpaired Student’s t test was used for two-group comparisons, and one-way ANOVA with an LSD post hoc test was used for multi-group comparisons.

**Fig. S2. Elevated SRPK3 was identified in HCM.**

**(A).** Schematical transcriptional analysis of SRPKs in human RNA-seq data including donor (n=39), HCM (n=106) myocardium (GSE36961).

(**B-C**) Transcriptional expression of SRPK1 and SRPK2.

(**D**) Positive correlation between levels of SRPK3 and different hypertrophic elements (TPM1) among HCM and control tissues, and correlation was analyzed using Spearman correlation analysis.

r and *p* values were shown. unpaired Student’s *t* test was performed.

**Fig. S3. Up-regulation of SRPK3 in human end-stage HF myocardium.**

(**A**) Summary of demographic data of normal and end-stage HF subjects used in this study. More information on individual subjects is provided in table S1.

(**B**) Immunoblot of LV tissues from 35 normal and 47 HF individuals after heart transplants or endomyocardial biopsy. Accordant augment of SRPK3 and Myh7 was indicated by blue arrowheads.

(**C**) Relative SRPK3 and Myh7 proteins in LV tissues from 35 normal and 47 HF individuals.

(**D**) Positive correlation between protein levels of SRPK3 and Myh7 among normal and end-stage HF subjects, and correlation was analyzed using Spearman correlation analysis.

(**E**) Dot plot image of individuals(n=3 for normal individuals and n=6 for HF patients) with definite cardiac hypertrophy, related to figure 1D.

r and p values of the Pearson correlation coefficient are shown. unpaired Student’s *t* test was performed.

**Fig. S4.** **Elevated SRPK3 was essentially enriched in cardiomyocytes, especially in nuclear localization.**

**(A)** Dot plot visualization of representative marker genes used to identify clusters. The color and size of dots indicate the relative average expression level in each population and the percentage of cells expressing the gene, respectively.

**(B)** Heatmap displaying the most differentially expressed genes in each major cell types.

(**C**) Cellular SRPK3 distribution by snRNA analysis in human hypertrophic hearts.

(**D**) Ang II induced cellular SRPK3 immunoblot in primary cardiomyocytes (CMs), cardiac fibroblasts (CFs), endothelial cells (ECs) and macrophage (Mφ) isolated from mouse heart.

(**E**) Ang II induced cellular Srpk3 mRNA in various cell types(n=4 for each group).

(**F**) Schematical map of hiPSC induced differentiation into cardiomyocytes and post hypertrophic model processing.

(**G**) The cellular fraction of hiPSC-CMs treated with Ang II(n=6) or not(n=6) was harvested for SRPK3 immunoblot analysis.

*p* values were shown. unpaired Student’s *t* test was performed.

**Fig. S5. Cardiac SRPK3 is not altered in physiological hypertrophy.**

(**A**) Schematic diagram of swim induced mouse model of physiological hypertrophy.

(**B**) M model echocardiography of mice.

(**C-F**) Echocardiography parameters of swim group(n=8) and rest group(n=8) including LV ejection fraction, LV fractional shortening, and LV end systolic/diastolic diameter of **B** were calculated.

(**G-H**) Representative WGA images and quantification of cross-sectional area of myocytes of swim group(n=8) and rest group(n=8).

(**I-J**) Immunoblot and relative levels of SRPK3 in hearts of swim group(n=6) and rest group(n=6).

(**K-L**) Immunoblot and relative levels of SRPK3 in hiPSC-CMs of control group(n=6) and IGF-1 incubation group(n=6).

(**M-N**) Relative SRPK3 transcription in hypertrophic myocardium and neonatal rat cardiomyocytes based on public data GSE56348(n=5 for each group) and GSE73896(n=4 for each group), respectively.

*p* values were shown. unpaired Student’s *t* test was performed.

**Fig. S6. Generation and verification of Srpk3^Tg/Tg^*-Myh6*-MerCreMer mice.**

(**A**) Schematical map showed Srpk3^Tg/+^ mice were crossed with *Myh6*-MerCreMer mice to generate Srpk3^Tg/Tg^*-Myh6*-MerCreMer (Srpk3 cTg) mice.

(**B**) Genotyping primers for gene identification of Srpk3 cTg offsprings, and (**C**) the routine PCR electrophoresis results from indicated mice.

(**D**) Relative Srpk3 mRNA in primary cardiomyocytes isolated from WT male(n=6) and female(n=6) or Srpk3 cTg male(n=6) and female(n=6) mice.

(**E-F**) Immunoblot and relative proteins of SRPK3 in primary cardiomyocytes isolated from WT male(n=6) and female(n=6) or Srpk3 cTg male(n=6) and female(n=6) mice.

(**G**) Body weight and echocardiography parameters including LV ejection fraction, LV fractional shortening, and LV end systolic/diastolic diameter were calculated in Myh6-cre(n=7) or C57BL/6J(n=7) mice subjected to mere corn oil and Myh6-cre(n=6) or C57BL/6J(n=6) mice subjected to tamoxifen injection.

(**H-I**) Immunoblot and relative proteins of SRPK3, BNP, Myh7 in WT or Srpk3 cTg mice.

*p* values were shown. Two-tailed Student’s t test was used for two-group comparisons, and one-way ANOVA with an LSD post hoc test was used for multi-group comparisons.

**Fig. S7. Exogenous SRPK3 extending drivers hypertrophic growth in hiPSC-CMs.**

(**A**) The rough procedure of Ad-SRPK3 transfection in hiPSC-CMs.

(**B**) Immunoblot verification of SRPK3 overexpression at different adenovirus viral titer.

(**C**) Relative natriuretic peptide A (*NPPA)* and natriuretic peptide B (*NPPB)* mRNA in cardiomyocytes following Ad-SRPK3 (50 MOI) (n=6) or CTR(n=6) infection.

(**D-F**) Immunoblot and relative proteins of SRPK3, Myh7 and BNP in whole cell lysate (WCL) after Ad-SRPK3 transfection at different adenovirus viral titer(n=6 for each group).

(**G-H**) Representative IF images of α-Actinin (red) in hiPSC-CMs after Ad-SRPK3 (50 MOI) transfection, and cross-sectional area was calculated (Ad-CTR, n=28; Ad-SRPK3, n=26).

(**I**). Representative IF images of α-Actinin (green) in hiPSC-CMs after SRPK3 or CTR administration. An intensity plot measured along the white rectangular box.

*p* values were shown. Two-tailed Student’s t test was used for two-group comparisons, and one-way ANOVA with an LSD post hoc test was used for multi-group comparisons.

**Fig. S8. Generation and verification of Srpk3^fl/fl^-*Myh6*-MerCreMer mice.**

(**A**) Schematical map showed cardiomyocyte-specific Srpk3 (Srpk3 cKO) mice were generated by mating Srpk3^fl/fl^ mice with *Myh6*-MerCreMer mice.

(**B**) Genotyping primers for gene identification of Srpk3 cKO offsprings, and (**C**) the routine PCR electrophoresis results from indicated mice.

(**D**) Immunoblot of SRPK3 and CRE in heart homogenates from WT(n=6) or Srpk3 cKO(n=6) mice.

(**E**) Heart weight/body weight (HW/BW) ratio and lung weight/tibia length (LW/TL) ratio were calculated(n=9 for sham groups; n=8 for TAC groups).

(**F-G**) Representative isolated adult mouse cardiomyocytes (AMCMs) from indicated mice, and cross-sectional area was calculated.

*p* values were shown. One-way ANOVA with an LSD post hoc test was used for multi-group comparisons.

**Fig. S9. Ang II incubation induced cardiomyocyte hypertrophy was attenuated by SRPK3 silencing.**

(**A**) The rough procedure of Ad-shSRPK3 transfection in hiPSC-CMs following Ang II treatment.

(**B**) Immunoblot verification of SRPK3 knockdown by Ad-shSRPK3.

(**C**) Relative *NPPA* and *NPPB* mRNA in cardiomyocytes(n=6 for each group).

(**D-E**) Immunoblot and relative proteins of SRPK3, Myh7 and BNP inside WCL in indicated groups(n=6 for each group).

(**F-G**) Representative IF images of α-Actinin (red) in Ang II treated hiPSC-CMs after Ad-shSRPK3 transfection, and cross-sectional area was calculated (PBS-shCTR, n=24; PBS-shSRPK3, n=28; Ang II-shCTR, n=27; Ang II-shSRPK3, n=30).

*p* values were shown. One-way ANOVA with an LSD post hoc test was used for multi-group comparisons.

**Fig. S10. SRPK3 controls mitochondrial metabolism and integrity.**

(**A**) The rough procedure of transcriptome in WT or Srpk3 cTg hearts at 6th week of tamoxifen induction.

(**B-C**) Heat map and volcano plot showed the differentially expressed genes (DEGs) between WT or Srpk3 cTg hearts.

(**D**). GSEA analysis showing the enrichment score of Fatty Acid Oxidation(FAO), cardiac muscle contraction and pyruvate metabolism gene sets in LV tissues.

(**E**) Heatmap showing differentially expressed mitochondrial inner membrane (IM), outer membrane (OM), and matrix genes in Srpk3 cTg and WT hearts at 6th week of tamoxifen injection.

(**F**) Relative mRNA expression of mitochondrial IM, OM and matrix genes in Srpk3 cTg and WT hearts at 6th week of tamoxifen injection(n=4 for each group).

(**G**) Relative mRNA expression of mitochondrial IM, OM and matrix genes in WT and Srpk3 cKO hearts(n=4 for each group).

(**H**) Immunoblot of mitochondrial IM, OM and matrix components in murine LV tissues from Srpk3 cTg and WT mice at 6th week of tamoxifen injection, and WT and Srpk3 cKO mice after TAC treatment for 4 weeks.

*p* values were shown. Unpaired Student’s *t* test was performed.

**Fig. S11. Genetic SRPK3 intervention governs mitochondrial dysfunction with no impact on mitophagy.**

(**A**) The ratio of mitochondrial DNA (mtDNA) to nuclear DNA (nDNA) was determined by RT-PCR in WT(n=9) or Srpk3 cTg(n=9) hearts.

(**B**) Representative TEM images of WT and Srpk3 cKO mice after TAC operation.

(**C**) Quantification of mitochondria-related parameters including Mitochondria/μm^2^ of Srpk3^fl/fl^ (n=7) and Srpk3 cKO(n=8), mitochondria with loss of cristae of Srpk3^fl/fl^(n=8) and Srpk3 cKO(n=9) and mitochondrial area from B. Mitochondria/μm^2^ refers to the average number of mitochondria; mitochondrial area refers to the ratio of mitochondrial area to image area (WT, n=231; Srpk3 cTg, n=228); mitochondria with loss of cristae refers to the percentage of defective mitochondria in individual samples.

(**D**) Quantification of basal respiration, ATP production-coupled respiration, maximal respiration and spare respiration capacity related to figure 3M in main document(n=6 for each group).

(**E-F**)OCR in hiPSC-CMs with or without SRPK3 silencing under PBS conditions, and quantification of mitochondrial respiration capacity related to figure 3N in main document(n=6 for each group).

(**G**) Immunoblot and relative proteins of Pink, Parkin, BNIP3L, LC3 in Srpk3 cTg and WT hearts at 6th week of tamoxifen injection(n=6 for each group).

(**H-I**) Representative fluorescence images of AMCMs from WT/Mito-Keima(n=6) or Srpk3 cTg/Mito-Keima(n=6) mice, the graph shows quantification of high (561nm/467nm) Keima signal area.

(**J-K**) Representative images are shown in WT/Mito-Keima(n=7) and Srpk3 cTg/Mito-Keima(n=6) mice, and high (561nm/467nm) signal areas of Mito-Keima dots/total microscopic areas are shown.

*p* values were shown. Two-tailed Student’s t test was used for two-group comparisons, and one-way ANOVA with an LSD post hoc test was used for multi-group comparisons.

**Fig. S12. Global RNA splicing analysis in Srpk3 cTg or WT mice.**

(**A**) Volcano plots showed changed alternative splicing events in Srpk3 cTg/ WT littermates’ hearts at 6th week of tamoxifen injection. The value range of -Log10 FDR and Inc level difference was listed in the figure.

(**B**) Volcano plots showed changed alternative splicing events in WT littermates’/6J background animals’ hearts. The value range of -Log10 FDR and Inc level difference was listed in the figure.

**Fig. S13. SRPK3 binds to and phosphorylates RPS3^Ser149^.**

(**A**) Schematic of the SRPK3 interaction discovery and lists of proteins.

(**B**) Subcellular localization of candidates in SRPK3 interaction discovery.

(**C**) Bubble chart of SRPK3 interacting candidate proteins by immunoprecipitation-mass spectrographic analysis.

(**D**) Clusters of orthologous groups in SRPK3 interacting candidate proteins.

(**E**) Analysis of Protein-Protein Interaction (PPI) networks in SRPK3 interacting candidate proteins. Srsf2 is marked in blue, and RPS3 in red.

(**F**) Molecular docking results.

(**G**) Exogenous Co-immunoprecipitation of SRPK3 and RPS3 was performed in Flag-RPS3 and HA-SRPK3 transfected HEK293T cells.

(**H**) SRPK3 was identified by LC/LC-MS in RPS3 96-152aa protein complex.

(**I**) Conservative analysis of Ser149 among multiple species.

**Fig. S14. RNA-Seq analysis upon RPS3 S149A mutant or WT cardiomyocytes following Ang II incubation.**

(**A**) Pie chart showing the composition of RPS3 binding RNAs in hiPSC-CMs.

(**B-C**) Heat map and volcano plot showed the differentially expressed genes (DEGs) between RPS3 mutant or WT cardiomyocytes following Ang II incubation.

(**D-E**) Gene Ontology analysis of molecular function for up-regulated and down-regulated genes in RPS3 mutant cardiomyocytes.

(**F**) GSEA plot showing up-regulation of RNA stabilization processing pathway under RPS3 mutant.

(**G**) The RNA stabilization-related genes in RPS3 S149A mutant cells compared to with WT(n=4 for each group).

(**H**) Venn diagrams showing the overlap of RPS3-binding repeats and up-/downregulated repeats in RPS3 S149A mutant cardiomyocytes.

(**I**) In RNA-FISH assays, RPS3 was colocalized with *PPARGC1A* mRNA in cardiomyocytes.

(**J**) Relative mRNA expression of *PPARGC1A* gene in RPS3 mutant(n=4) or WT(n=4) cardiomyocytes.

*p* values were shown. Unpaired Student’s *t* test was performed.

**Fig. S15. Genetic conversion of** **mtRNA metabolism impairs hypertrophic growth in SRPK3 overexpressing cardiomyocytes.**

(**A**) Sequence of human RPS3 gene encoding part of the regulatory domain and Serine 149 mutation.

(**B**) Representative Sanger sequencing chromatogram for editing the RS domain of human RPS3.

(**C**) Schematic of Ad-SRPK3 transfected hiPSC-CMs with RPS3^Ser149Ala^ or RPS3^ΔRS^.

(**D-E**) Relative mRNA expression of *NPPA*, *NPPB*, *PPARGC1A* gene in indicated cardiomyocytes(n=4 for each group).

(**F**) RT-PCR analysis for mitochondrial genes between Ad-CTR(n=3) and SRSF2 WT(n=3) , SRSF2^ΔRS^(n=3) cardiomyocytes subjected to Ad-SRPK3. The middle panels represent the schematic diagram of indicated AS exons. Right panels show the quantification of percent spliced in (PSI). 3 independent biological replicates are presented.

(**G-H**) Immunoblot and relative proteins of BNP, Myh7, PGC1α and OXPHOS complex in genetic editing hiPSC-CMs(n=6 for each group).

(**I**) The OCR in hiPSC-CMs of Ad-CTR, RPS3 Ser149A, SRSF2^ΔRS^, WT with Ad-SRPK3.

(**K-L**) Representative IF images of α-Actinin (red) in genetic editing cardiomyocytes after Ad-shSRPK3 transfection, and cross-sectional area was calculated (Ad-CTR, n=38; Ad-SRPK3+WT cells, n=36; Ad-SRPK3+ hiPSC-CMs with RPS3^Ser149Ala^, n=20; Ad-SRPK3+ hiPSC-CMs with SRSF^ΔRS^, n=19).

(**J**) Quantification of basal respiration, ATP production-coupled respiration, maximal respiration and spare respiration capacity from J (n=4 for each group).

*p* values were shown. One-way ANOVA with an LSD post hoc test was used for multi-group comparisons.

**Fig. S16. Knockdown of SRPK3 slowed down cardiac hypertrophy and mitochondrial defects in mice.**

(**A**) Immunoblot of SRPK3 in heart, liver, and kidney homogenates from AAV9-cTnT-shCTR or AAV9-cTnT-shSRPK3 treated mice.

(**B**) Echocardiography parameters of male subjected with AAV9-cTnT-shCTR(n=5) and AAV9-cTnT-shSRPK3(n=5 for LVEF, n=6 for LVFS) and female mouse subjected with AAV9-cTnT-shCTR(n=6) and AAV9-cTnT-shSRPK3(n=5) including LV ejection fraction, LV fractional shortening.

(**C**) heart weight/tibia length (HW/TL) ratio and cross-sectional area of male subjected with AAV9-cTnT-shCTR(n=5) and AAV9-cTnT-shSRPK3(n=6) and female mouse subjected with AAV9-cTnT-shCTR(n=6) and AAV9-cTnT-shSRPK3(n=5).

(**D-E**) Immunoblot and relative proteins of BNP, Myh7, PGC1α and OXPHOS complex in TAC-treated heart tissue homogenate with or without SRPK3 silencing intervention (n = 6 for each group).

(F) Immunoblot and relative proteins of p-RPS3, RPS3, p-SRSF2 and SRSF2 in TAC-treated heart tissue homogenate with or without SRPK3 silencing intervention(n = 6 for each group). *p* values were shown. One-way ANOVA with an LSD post hoc test was used for multi-group comparisons.

**Supplementary Tables**

**Table S1: Baseline characteristics of the study participants. Related to Figure 1.**

| Characteristics | Normal  (n=35) | End-stage HF  (n=47) |
| --- | --- | --- |
| Sex (M/F) | 19M/16F | 28M/19F |
| Age, year | 58.3±10.1 | 53.7±9.6 |
| BMI, kg/m^2^ | 23.68±4.8 | 24.82±3.7 |
| FPG, mmol/L | 5.4±0.6 | 6.1±1.8 |
| HR (/min) | 73±12.2 | 76±9.1 |
| Triglycerides, mmol/L | 1.2 (0.9-1.9) | 1.6 (1.2-2.4) |
| Cholesterol, mmol/L | 4.2±0.8 | 4.7±1.2 |
| HDL cholesterol, mmol/L | 1.2±0.2 | 1.1±0.3 |
| LDL cholesterol, mmol/L | 2.4±1.0 | 2.5±0.8 |
| LVM (g)/ BSA (m^2^) | 118±6.9 | 122±8.1 |
| NT-proBNP (pg/mL) | 169±42 | 2417±529*** |
| Blood Pressure |  |  |
| SBP (mmHg) | 126±33.6 | 153±40.3* |
| DBP (mmHg) | 72±12.2 | 73±9.8 |
| Echocardiography |  |  |
| LVEF% | 65±6.1 | 48±9.7*** |
| RAD (mm) | 36±6.2 | 43±9.1** |
| LAD (mm) | 33±7.2 | 43±8.3** |
| LVDD (mm) | 48±5.9 | 54±6.7* |
| RVD (mm) | 20±6.9 | 26±8.1* |
| IVSD (mm) | 10±1.6 | 11±1.4 |
| LVPWT (mm) | 10±1.2 | 10±1.3 |

BMI, body mass index; FPG, fasting plasma glucose; HR, heart rate; HDL, high density lipoprotein; LDL, low density lipoprotein; LVM, Left ventricular mass; BSA, body surface area; NT-proBNP, N-terminal pro-brain natriuretic peptide; SBP, systolic blood pressure; DBP, diastolic blood pressure; LVEF, left ventricular ejection fraction; RAD, right atrium diameter; LAD, left atrium diameter; LVDD, left ventricular end diastolic diameter; RVD, right atrium diameter; IVSD, interventricular septal thickness at diastole; LVPWT, left ventricular posterior wall thickness. Data are means ± SEM. *p<0.05, ** p<0.01, *** p<0.001 vs normal individuals.

**Table S2:** **Heart size and function in wild-type littermates and *Srpk3* cTg mice. Related to Figure 2.**

|  | WT littermates | *Srpk3* cTg |
| --- | --- | --- |
| Gravimetry | | |
| Body weight (g) | 28.7±0.6 | 28.3±0.9 |
| Heart weight (mg) | 112.4±8.8 | 231.7±12.1*** |
| Lung weight (mg) | 141.3±8.9 | 143.6±7.9 |
| Parameters of heart function | | |
| Heart rate (min^-1^) | 462±17.2 | 461±18.1 |
| LVEDP (mmHg) | 6±1.1 | 11±1.0** |
| LVESP (mmHg) | 92±3 | 98±4* |
| LVIDs (mm) | 2.2±0.15 | 5.1±0.21*** |
| LVIDd (mm) | 2.7±0.17 | 4.8±0.19*** |
| LVEF (%) | 76±8 | 43±7*** |
| LVFS (%) | 40±1.7 | 22±1.8*** |
| Cardiac output (mL/min) | 12.2±0.9 | 7.8±1.6** |

Cardiac functional parameters in WT littermates and *Srpk3* cTg mice at the sixth week of tamoxifen injection were evaluated. LVEDP, LV end-diastolic pressure; LVESP, LV end-systolic pressure; LVIDs, LV end-systolic diameter; LVIDd, LV end-diastolic diameter; LVEF, LV ejection fraction; LVFS, LV fraction shortening; Data are means ± SEM from 8-10 WT and 9-12 *Srpk3* cTg mice per genotype. *p<0.05, ** p<0.01, *** p<0.001 vs WT littermates.

**Table S3:** **Heart size and function in wild-type littermates and *Srpk3* cKO mice following Sham or TAC surgery. Related to Figure 2.**

|  | WT, Sham | *Srpk3* cKO, Sham | WT, TAC | *Srpk3* cKO, TAC |
| --- | --- | --- | --- | --- |
| Gravimetry | | | | |
| Body weight (g) | 27.8±0.7 | 28.1±0.6 | 28.2±0.8 | 27.9±0.8 |
| Heart weight (mg) | 116.1±9.4 | 115.8±8.4 | 251.1±10.2*** | 207.3±8.9^##^ |
| Lung weight (mg) | 149.2±10.1 | 147.3±9.6 | 214.3±15.1*** | 209.8±14.3^#^ |
| Parameters of heart function | | | | |
| Heart rate (min^-1^) | 457±18.9 | 466±9.7 | 469±19.6 | 467±10.7 |
| LVEDP (mmHg) | 7.1±1.1 | 6.8±1.0 | 11.7±0.9** | 9.7±1.0^##^ |
| LVESP (mmHg) | 89±5 | 91±6.1 | 102±13.9* | 96±11.2^#^ |
| LVIDs (mm) | 2.3±0.16 | 2.1±0.19 | 6.1±0.96*** | 3.8±0.15^###^ |
| LVIDd (mm) | 2.6±0.17 | 2.9±0.21 | 5.1±0.36*** | 4.6±0.28^#^ |
| LVEF (%) | 77±6 | 79±7 | 34±5*** | 53±8^##^ |
| LVFS (%) | 41±2.1 | 43±1.6 | 23±3.3*** | 37±1.9^##^ |
| Cardiac output (mL/min) | 13.1±0.8 | 12.7±1.3 | 7.6±1.7** | 10.9±1.3^###^ |

WT littermates and *Srpk3* cKO mice underwent sham or transverse aortic constriction (TAC) surgery. Measurements were performed after 4 weeks. LVEDP, LV end-diastolic pressure; LVESP, LV end-systolic pressure; LVIDs, LV end-systolic diameter; LVIDd, LV end-diastolic diameter; LVEF, LV ejection fraction; LVFS, LV fraction shortening; Data are means ± SEM. *p<0.05, ** p<0.01, *** p<0.001 vs WT littermates following Sham, and ^#^p<0.05, ^##^ p<0.01, ^###^ p<0.001 vs WT littermates following TAC.

**Table S4:** **Genomic sites targeted by sgRNAs designed in the study.**

| Locus | Site | Sequences targeted by tested sgRNAs |
| --- | --- | --- |
| RPS3 Ser149 | 5′ upstream | 1-5′ GGCCAAAGGCTGCGAGGTTGTGG 3′ |
|  |  | 2-5′ GCTGCGAGGTTGTGGTGTCTGGG 3′ |
|  |  | 3-5′ TGAGTTTCTTACTCGGGAGCTGG 3′ |
|  | 3′ downstream | 1-5′ TGATGATCCACAGCGGAGACCTG 3′ |
|  |  | 2-5′ CCGAGGACAGAGGGCTAAATCCA 3′ |
|  |  | 3-5′ AGTGTGCTGGGCATCAAGGTGAA 3′ |
| SRSF2 exon 32-42 | 5′ upstream | 1-5′ TCACGACAAGCGCGACGCTGAGG 3′ |
|  |  | 2-5′ TACTACGTTGACACTGCTGTGCG 3′ |
|  |  | 3-5′ CTTGGACCTTCGTGCGGATCTGG 3′ |
|  | 3′ downstream | 1-5′ CTTACTCGGGAGCTGGCTGAAGA 3′ |
|  |  | 2-5′ CCTGCTATGGTGTGCTGCGGTTCA 3′ |
|  |  | 3-5′ CTTACTCGGGAGCTGGCTGAAGAT 3′ |

**Table S5:** **Primers used to amplify genomic regions with putative off-target effects of sgRNAs used in the study.**

| Locus | sgRNA | Genomic region | Primers |
| --- | --- | --- | --- |
| RPS3 | 5′ upstream  sgRNA1 | chr17:76200940 | 5′ GCGCATAGCGTGCGTGCTTATAG 3′ |
|  |  | chrX:10085296 | 5′ GTACGAGAAGCCCGACGCTGAGG 3′ |
|  |  | chr3:145878819 | 5′ TCGCGAGAAGCGCGGCGCTGCGG 3′ |
|  |  | chr13:33337047 | 5′ CTTGTCTTGAAAGCAAACAAGGG 3′ |
|  | 3′ downstream  sgRNA1 | chr14:61114367 | 5′ CTAGTCTTGGAAGCGAACAA AGG 3′ |
|  |  | chr1:218933623 | 5′ CTTGTCTTGAAAGTGAAAAAAGG 3′ |
|  |  | chr9:139266498 | 5′ ACGAGAACGATGACGAGTGCTGG 3′ |
|  |  | chr5:126455760 | 5′ AGGCCATGTCCCCAGTAGCAAGG 3′ |
| SRSF2 | 5′ upstream  sgRNA1 | chr2:85872016 | 5′ AGACAAAGGCTGTGAGGTTAAGG 3′ |
|  |  | chr18:21253286 | 5′GGTCAAAGGCTGCAAAGTTTCGG 3′ |
|  |  | chr1:2769812 | 5′ TGCCAAGTGCTGCAAGGTTGTGA 3′ |
|  |  | chr6:7339388 | 5′ CACCACGACCTCACAGCCTT 3′ |
|  | 3′ downstream  sgRNA1 | chr12:62415660 | 5′ GCTGTGAGGTCATGGTGTCTAGA 3′ |
|  |  | chr20:9457199 | 5′ AGGGGAAAGTTTGTCACTGAAGA 3′ |
|  |  | chr13:32937466 | 5′ TAGTGCAGATACCCAAAAAGTGA 3′ |
|  |  | chr11:75112701 | 5′ GTGAGAAGGGCCGGCGGATTCGA 3′ |

**Table S6:** **Heart size and function in AAV9-cTnT-shSRPK3 or AAV9-cTnT-shCTR treated mice following Sham or TAC surgery for 4 weeks. Related to Figure 7.**

|  | shCTR, Sham | shSRPK3, Sham | shCTR, TAC | shSRPK3, TAC |
| --- | --- | --- | --- | --- |
| Gravimetry | | | | |
| Body weight (g) | 27.1±0.8 | 26.9±0.6 | 27.5±0.8 | 27.4±0.9 |
| Heart weight (mg) | 131±8.9 | 121±9.7 | 248±10.1*** | 203±9.3^#^ |
| Lung weight (mg) | 143.6±9.4 | 141.9±10.5 | 209.3±11.1** | 188.3±10.9^##^ |
| Parameters of heart function | | | | |
| Heart rate (min^-1^) | 462±19.6 | 463±14.4 | 459±18.3 | 465±16.4 |
| LVEDP (mmHg) | 7.3±1.0 | 7.2±1.2 | 12.1±1.0*** | 9.5±1.1^##^ |
| LVESP (mmHg) | 90±6.1 | 88±5.9 | 105±11.3** | 93±10.7^#^ |
| LVIDs (mm) | 2.2±0.11 | 2.3±0.17 | 5.8±0.61*** | 4.2±0.19^##^ |
| LVIDd (mm) | 2.7±0.11 | 3.1±0.18 | 4.9±0.28** | 3.7±0.21^#^ |
| LVEF (%) | 79±5 | 78±6 | 32±6*** | 61±9^###^ |
| LVFS (%) | 39±2.3 | 41±1.9 | 24±2.9*** | 36±3.3^###^ |
| Cardiac output (mL/min) | 14.1±0.9 | 13.6±1.1 | 9.1±1.5** | 11.5±1.7^##^ |

Mice underwent sham or transverse aortic constriction (TAC) surgery were treated with AAV9-cTnT-shSRPK3 or AAV9-cTnT-shCTR. Measurements were performed after TAC for 4 weeks. LVEDP, LV end-diastolic pressure; LVESP, LV end-systolic pressure; LVIDs, LV end-systolic diameter; LVIDd, LV end-diastolic diameter; LVEF, LV ejection fraction; LVFS, LV fraction shortening; Data are means ± SEM. *p<0.05, ** p<0.01, *** p<0.001 vs mice following Sham, and ^#^p<0.05, ^##^ p<0.01, ^###^ p<0.001 vs mice following TAC.

**Table S7:** **Key resources.**

| Reagents | No. | Suppliers | RRID |
| --- | --- | --- | --- |
| SRPK3 | PA5103255 | Thermo Fisher Scientific | AB_2852618 |
| SRPK3 | M6568 | Sigma-Aldrich | AB_2194861 |
| β-MHC | A7564 | ABclonal | — |
| BNP | A2197 | ABclonal | — |
| α-SMA | ab5694 | Abcam | — |
| OXPHOS | ab110413 | Abcam | AB_2629281 |
| BNIP2 | PA549988 | Thermo Fisher Scientific | AB_2635441 |
| POLG2 | A14504 | ABclonal | AB_2761378 |
| NME4 | A8350 | ABclonal | AB_2770602 |
| DRAS2 | A7813 | ABclonal | AB_2769114 |
| CHCHD6 | A12911 | ABclonal | AB_2759756 |
| SRSF2 | A3635 | ABclonal | AB_2765195 |
| DYKDDDDK Tag | 8146 | Cell Signaling Technology | AB_10950495 |
| HA-Tag | 3724S | Cell Signaling Technology | AB_1549585 |
| RPS3 | A11161 | ABclonal | AB_2758439 |
| phos(Ser149)-RPS3 | — | ABclonal | — |
| GST Tag | 136700 | Thermo Fisher Scientific | AB_2533028 |
| His | MA121315 | Thermo Fisher Scientific | AB_2536989 |
| Lamin B1 | ab8982 | Abcam | AB_1640627 |
| α-Actinin | 69758 | Cell Signaling Technology | AB_2799767 |
| PGC1α | 4259 | Cell Signaling Technology | AB_2268435 |
| mCherry | PA5143614 | Thermo Fisher Scientific | AB_2942843 |
| GFP | 332600 | Thermo Fisher Scientific | AB_2533111 |
| BPHL | 17974-1-AP | Proteintech | AB_2274994 |
| ACSL | NB110-99585 | Novus | AB_1502544 |
| OGDHL | A15475 | ABclonal | AB_2762873 |
| GSTK1 | A9667 | ABclonal | AB_2863755 |
| OXA1L | A6300 | ABclonal | AB_2766905 |
| MTFP1 | A7110 | ABclonal | AB_2767665 |
| PINK | PA586941 | Thermo Fisher Scientific | AB_2803698 |
| Parkin | PA532539 | Thermo Fisher Scientific | AB_2550005 |
| BNIP3L | PA529385 | Thermo Fisher Scientific | AB_2546861 |
| LC3 | 12513 | Cell Signaling Technology | AB_2797941 |
| Myc Tag | 2040 | Cell Signaling Technology | AB_2148465 |
| Goat anti-Rabbit IgG Secondary Antibody | A16098 | Thermo Fisher Scientific | AB_2534772 |
| Secondary Antibody, Alexa Fluor™ 488 | A11001 | Thermo Fisher Scientific | AB_2534069 |
| Secondary Antibody, Alexa Fluor™ 568 | A-11004 | Thermo Fisher Scientific | AB_2534072 |
| SYBR Green Master | 470751001 | Roche | — |

**Table S8:** **Primers used for the RT-qPCR.**

| Gene | Species | Primers |
| --- | --- | --- |
| *NPPA* | Homo | CACCGTGAGCTTCCTCCTTT  TCGATCAGAGGAGTCCCAGG |
| *NPPB* | Homo | CTTTCCTGGGAGGTCGTTCC  CTGTAACCCGGACGTTTCCA |
| *PPARGC1A* | Homo | GCTCTTTTGGCAGAAGGCATTT  GCTTGACTGGGATGACCGAA |
| *AXIN2* | Homo | GATGAGCCCCTGCTGACTTG  TCCCACTGAGTCTGGAATCTCT |
| *E2F1* | Homo | ACAAGGCCCGATCGATGTTT  CTGCAGAGACAAGGTGAGCA |
| *ZAR1* | Homo | GGAGGTGTGGACGAAGAAGG  CGTCTACTCCATCAGCGCAT |
| *TENT5C* | Homo | GTGAAGGTTTGCACGGACAC  ATGTTTTGTTCCGCCAGCAC |
| *TENT5A* | Homo | AAAGCTCCAGAGACACGAGC  GCGCTCAGACAGCAAATAGC |
| *RBM24* | Homo | AGATGCACACGACCCAGAAG  TAGTGGGCAGGTATCCCGAA |
| *GDNF* | Homo | CCCTCATGTCTTCACGGGAC  AAGAGTTCGCAATCCTGGGG |
| *RBM38* | Homo | AGAAGGACACCACGTTCACC  CCATGGTCACGAAGCCGTAG |
| *Nppa* | Mouse | TACCAGGGAGCATCTTTGGC  AGGGACACCCATCGTAGTGA |
| *Nppb* | Mouse | TTTGGGCTGTAACGCACTGA  TTGTGGCAAGTTTGTGCTCC |
| *Myh7* | Mouse | AGCATTCTCCTGCTGTTTCCT  TCCGGTGATGAGGATGGACT |
| *Acta2* | Mouse | GGCATCCACGAAACCACCTA  TTCCTGACCACTAGAGGGGG |
| *Col1a1* | Mouse | TTCTCCTGGCAAAGACGGAC  CCATCGGTCATGCTCTCTCC |
| *Tgfb1* | Mouse | ACTGGAGTTGTACGGCAGTG  GGGGCTGATCCCGTTGATTT |
| *Ndufv1* | Mouse | ATGTGTGACGCACTTCCTGT  CTGCTTCATAGGCCTCTCGG |
| *Ndufs1* | Mouse | CCAAGTCGTCTTCTCCGCTT  CCAGCCCTTCATTACAGGCA |
| *Sdhd* | Mouse | GTGACCTTGAGCCCTCGAAA  GCTGGTCCTGGAGAAATGCT |
| *Uqcrfs1* | Mouse | GAACGAAAGGTCGTCCCTGT  AAGGGTCGCTTCACATCCAG |
| *Uqcrc1* | Mouse | GGCTAACCCAGACAACGTGA  CAGGAGACTGCGACCAATGT |
| *Cox5a* | Mouse | TGTCTGTTCCATTCGCTGCT  AACCGTCTACATGCTCGCAA |
| *Cox7c* | Mouse | ATTTCTTCCGCCTTCCGTGT  CCGCCACTTGTTTTCCACTG |
| *Atp5c1* | Mouse | ATTTAGCGAGACCGAGGACG  GAACTTGGATCCATTGCGGC |
| *Atp5a1* | Mouse | TCCGAGAAGCTGCAAGGATG  TGGACCGCTTCTGACCAATC |
| *Srpk3* | Mouse | TCACGGGAGTTCTTCAACCG  TCTCATCCTGTCATGGGGGT |
| *Acsl1* | Mouse | AGTCAGCTGTCGGATGGCAC  AAACCCCATGAGGGTGTTGG |
| *Bphl* | Mouse | ATCCTCCTGCTTCCTGGGAT  TCGATCTGGGGGTCTGGAAT |
| *Cisd1* | Mouse | TCCCCGGTTCTAGATGTGGT  ACCCGGGCAAGTTTAAGGAG |
| *Ogdhl* | Mouse | TTTCCACAACACAGCCCAGT  GAATCAGCCGCTGGAAGCTA |
| *Dglucy* | Mouse | TCAAACCAACATTCAGCAGGAC  AGAGCGAAGACAGGACCTCA |
| *Gstk1* | Mouse | TGGATGCGTGTATGGTCTCG  TAGCAACTCCAAGCGGTCAG |
| *Oxa1l* | Mouse | CTTCTGCGACTCCTACGACC  CCAGTTCTGTGAAGCTCGGT |
| *Etfdh* | Mouse | CCGTACCTGAGTCCATTCCG  ATTGCCGTGTGGGTACCTTT |
| *Mtfp1* | Mouse | TTGTGCTGAGTGGGAACGAA  TTTCCCTAAGTCGCTCAGCC |
| *mtDNA* | Mouse | CTCTTATCCACGCTTCCGTTACG  GATGGTGGTACTCCCGCTGTA |
| *nDNA* | Mouse | GACATAAGGTTAGCAGCTAAAGGATCA  TCTCCGATTAACCAGGCTAGCTAT |
